# The effects of telerehabilitation on physiological function and disease symptom for patients with chronic respiratory disease: a systematic review and meta-analysis

**DOI:** 10.1186/s12890-024-03104-8

**Published:** 2024-06-28

**Authors:** Yue Dai, Hao Huang, Yuchen Zhang, Na He, Min Shen, Hong Li

**Affiliations:** 1 Department of Emergency Medicine, West China Hospital, Sichuan University/West China School of Nursing, Sichuan University, Chengdu, Sichuan China; 2 Institute of Disaster Medicine, Sichuan University, Chengdu, Sichuan China; 3 Nursing Key Laboratory of Sichuan Province, Chengdu, Sichuan China; 4 West China School of Nursing, Sichuan University, Sichuan, China; 5 Department of Nursing, West China Tianfu Hospital, Sichuan University, Chengdu, Sichuan China; 6 Department of Day Surgery, West China Hospital, West China Tianfu Hospital, Sichuan University, Chengdu, Sichuan China

**Keywords:** Chronic respiratory diseases, Telerehabilitation, Meta-analysis

## Abstract

**Objective:**

To compare the impact of telerehabilitation versus conventional rehabilitation on the recovery outcomes of patients with chronic respiratory disease (CRD).

**Methods:**

The Cochrane Library, MEDLINE, Web of Science and Embase were searched to collect randomized controlled trials (RCTs) on telerehabilitation for the rehabilitation of patients with chronic respiratory system diseases since the establishment of the database to November 14, 2023. Two researchers independently screened the literature and extracted valid data according to the inclusion criteria. The quality assessment of included studies was conducted individually by using the RoB 2(Risk of Bias 2) tool, followed by meta-analysis using RevMan5.3 software.

**Results:**

Based on inclusion and exclusion criteria, 21 RCTs were included, comprising 3030 participants, with 1509 in the telerehabilitation group and 1521 in the conventional rehabilitation group. Meta-analysis results indicated that compared to conventional rehabilitation, video conference-based telerehabilitation demonstrated significant improvements in short-term (≤ 6 months) outcomes, including 6-min walk distance (6MWD) (MD = 7.52, 95% CI: 2.09, 12.94), modified Medical Research Council Dyspnea Scale (mMRC) (MD = -0.29, 95% CI: -0.41, -0.18), COPD assessment test (CAT) (MD = -1.77, 95% CI: -3.52, -0.02), HADS (MD = -0.44, 95% CI: -0.86, -0.03), and St. George’s Respiratory Questionnaire (SGRQ’s) activity, impact, and symptom scores. In the long term (> 6 months), although improvements persisted in 6WMD [MD = 12.89, 95% CI (-0.37, 26.14)], mMRC [MD = -0.38, 95% CI (-0.56, -0.21)], CAT [MD = -1.39, 95% CI (-3.83, 1.05)], Hospital anxiety and depression scale (HADS) [MD = -0.34, 95% CI (-0.66, -0.03)], and SGRQ’s Activity, Impact, and Symptom scores between intervention and control groups, statistically significant differences were observed only for mMRC and HADS. Without considering time factors, the intervention group exhibited some improvement in FEV1% predicted and the forced expiratory volume in the first one second (FEV1)/ forced vital capacity (FVC) (%) without statistical significance compared to the control group.

**Conclusion:**

Telerehabilitation therapy demonstrates short-term benefits in enhancing patients’ daily activity capacity, improving respiratory function, and enhancing mental health status, thereby improving patients’ quality of life. However, further high-quality, large-sample RCTs are required to ascertain its long-term effectiveness conclusively.

**Trial registration:**

This study protocol was approved and registered in PROSPERO: CRD 42024509154.

**Supplementary Information:**

The online version contains supplementary material available at 10.1186/s12890-024-03104-8.

## Introduction

Chronic Respiratory Diseases (CRD) represent a significant public health issue worldwide, encompassing conditions such as Chronic Obstructive Pulmonary Disease (COPD), bronchial asthma, bronchiectasis, interstitial lung diseases, obstructive sleep apnea syndrome, and lung cancer. CRD exhibit substantial morbidity, mortality, and disability rates. Global Burden of Disease studies suggest that CRD affects approximately 545 million individuals worldwide, constituting 7.4% of the global population [1]. Findings from the 2018 China Pulmonary Health (CPH) Study reveal that COPD’s overall prevalence among individuals aged 20 and above in China stands at 8.6%, with nearly 100 million patients nationwide. Notably, prevalence rates among males (11.9%) significantly surpass those among females (5.4%), with prevalence escalating with age. Among individuals aged 40 and above, COPD prevalence skyrockets to 13.7% [2]. Furthermore, according to the latest China Disease Burden Report, COPD ranks as the third leading cause of death among Chinese residents with a mortality rate of 68 per 100,000 [3].

CRD can result in debilitating symptoms, including dyspnea, fatigue, anxiety, depression, fear. It also impairs exercise tolerance, daily functioning, reduces quality-of-life, and escalates the risk of hospitalization and mortality, imposing substantial financial burdens on healthcare systems, amounting to billions of dollars annually. Among these, COPD accounts for 56% of the costs associated with CRD, serving as the most common cause of mortality from chronic respiratory system diseases [4].

Telerehabilitation (TR) refers to the provision of online medical and health services to returning home or home-based patients through technological means such as the internet, big data, and cloud computing. It offers physical therapy, speech therapy, remote monitoring, and consultations. TR provides a novel approach to pulmonary rehabilitation for CRD patients. It not only meets their medical service needs and reduces healthcare costs but also enhances the accessibility of service offerings. It addresses challenges faced in pulmonary rehabilitation such as transportation and distance barriers, thereby offering more choices for improving healthcare and pulmonary rehabilitation services. However, there are still some obstacles to participation in TR, including severe shortages of programs due to reasons such as patients’ lack of knowledge, insufficient funds, exacerbation of disease progression, transportation issues, and inadequate institutional support [5], which prevent patients from completing TR.

Previous meta-analyses have highlighted both the advantages and disadvantages of various intervention measures. However, these studies are not without limitations, including inadequate sample sizes [6] and a lack of observation regarding their effects on depression and anxiety [7]. Furthermore, with the rapid advancement of technology and the widespread application of telerehabilitation, an updated review is needed to assess the latest evidence and draw more robust conclusions. Hence, we conducted an updated meta-analysis based on randomized controlled trials (RCTs), incorporating a greater number of original studies, expanding the sample size, and consequently enhancing the effectiveness of the tests, eventually offering novel perspectives for clinical decision-making. In line with the “evidence-based research” framework, we have reviewed all systematic reviews on this topic to ensure that our study builds on the existing body of evidence and addresses the identified gaps [8–12]. This approach ensures the relevance and necessity of our review in contributing valuable insights to the ongoing discourse on telerehabilitation for CRD patients.

## Methods

This meta-analysis followed the guidelines outlined in the Cochrane Handbook for the Systematic Review of Interventions (for details, see at http://training.cochrane.org/handbook), as well as the Preferred Reporting Items for Systematic Review and Meta-Analyses for reporting it [13]. This study protocol was approved and registered in PROSPERO (CRD 42024509154).

### Inclusion and exclusion criteria

#### Study type

Parallel group randomized controlled trials (RCTs).

### Study participants

1. Age ≥ 18 years;
2. Patients diagnosed with CRD such as COPD, bronchiectasis, and interstitial lung disease;
3. Patients would have no major physical disabilities, could move around independently, and could participate in rehabilitation exercises and activities via remote methods.

### Intervention measures

Experimental group: remote pulmonary rehabilitation, such as telemedicine video consultation, Virtual Autonomous Physiotherapist Agent, video-guided exercises, etc.

Control group: standard care (Traditional exercise rehabilitation does not rely on remote technology).

### Outcome indicators

Based on the definition of CRD and the manifestation of rehabilitation effects, the following primary outcome measures were selected from both physiological function and disease symptom perspectives: 6-min walk test, St. George’s Respiratory Questionnaire (SGRQ), and modified Medical Research Council Dyspnea Scale (mMRC). Additionally, COPD Assessment Test (CAT), Hospital Anxiety and Depression Scale (HADS), and pulmonary function tests were chosen as secondary outcome measures to observe the rehabilitation effects of the two intervention methods on CRD patients.

The distance covered by the 6-min patient walking (6MWD) is shown as the results of the 6-min walk test.

### Exclusion criteria

1. The illness does not fall under the category of chronic respiratory disease;
2. Inaccessible study data;
3. non-RCTs, such as observational studies, case series and reviews.

### Retrieval strategies

According to the PICOS principal, we adopted mesh terms and free keywords in the search strategy.

1. Population (P): Patients diagnosed with CRD such as COPD, bronchiectasis, and interstitial lung disease.
2. Intervention (I): remote pulmonary rehabilitation, such as telemedicine video consultation, Virtual Autonomous Physiotherapist Agent, video-guided exercises, etc.
3. Comparison (C): standard care (Traditional exercise rehabilitation does not rely on remote technology).
4. Outcome (O): 6-min walk test, St. George’s Respiratory Questionnaire (SGRQ), modified Medical Research Council Dyspnea Scale (mMRC), COPD Assessment Test (CAT), Hospital Anxiety and Depression Scale (HADS), and pulmonary function tests.
5. Study design (S): randomized clinical trials (RCTs).

Computer searches were conducted in The Cochrane Library, MEDLINE, Web of Science, and Embase databases for studies on remote pulmonary rehabilitation since the establishment of the databases to November 14, 2023. English search Medical Subject Headings included: “Telemedicine”[MeSH Terms] AND (“Lung Diseases, Interstitial”[MeSH Terms] OR “Bronchiectasis”[MeSH Terms] OR “Pulmonary disease, chronic obstructive”[MeSH Terms]). The detailed search strategy is provided in Supplementary Material 1.

### Literature screening and data extraction

Two reviewers rigorously searched the literature according to the inclusion and exclusion criteria. All identified studies were managed using Endnote software version X9, with the retrieved documents imported into EndNote X9. Duplicate publications and non-English literature were excluded, and studies preliminarily meeting the criteria were screened based on titles or abstracts, with their full texts downloaded. Following full-text reading, original studies meeting the requirements for this systematic review were selected. Information was extracted from the literature and cross-checked, and units of measurement were standardized. In cases of disagreement, a third researcher was consulted for collective decision-making. Extracted information primarily included titles, first authors, publication years, countries, study types, sample sizes, and gender distributions in the intervention and control groups, intervention methods, intervention durations, and outcome measures.

### Assessment of bias risk in included studies

Two researchers independently assessed the risk of bias in the eligible studies using a bias assessment tool recommended in the Cochrane Handbook for Systematic Reviews of Interventions version 6.3, Chapter 8: Assessing risk of bias in a randomized trial, the Cochrane risk-of-bias tool for randomized trials (RoB 2), and the results were cross-validated. The risk of bias assessment involved the following seven domains: generation of random sequence (selection bias), allocation concealment (selection bias), blinding of participants and operators (performance bias), and blinding of outcomes assessment (detection bias), integrity of outcome data (attrition bias), selective reporting (reporting bias), and other sources of bias (other bias).

### Statistical methods

The meta-analysis was performed with RevMan (Version 5.3. Copenhagen: The Nordic Cochrane Centre, The Cochrane Collaboration, 2014). The magnitude of the effect of each study was calculated by the weighted mean difference (WMD) of the 95% confidence interval (CI) briefly. A *p*-value of < 0.05 was considered statistically significant unless otherwise specified. In addition, the heterogeneity was quantified using the Q-test and the *I*^2^ statistic. When *p* > 0.1 and *I*^2^ < 50%, a fixed-effect model was applied; otherwise, a random-effects model was used. If the heterogeneity was high, further analysis of the heterogeneity sources was performed.

## Results

### Literature search results

**Fig. 1:**
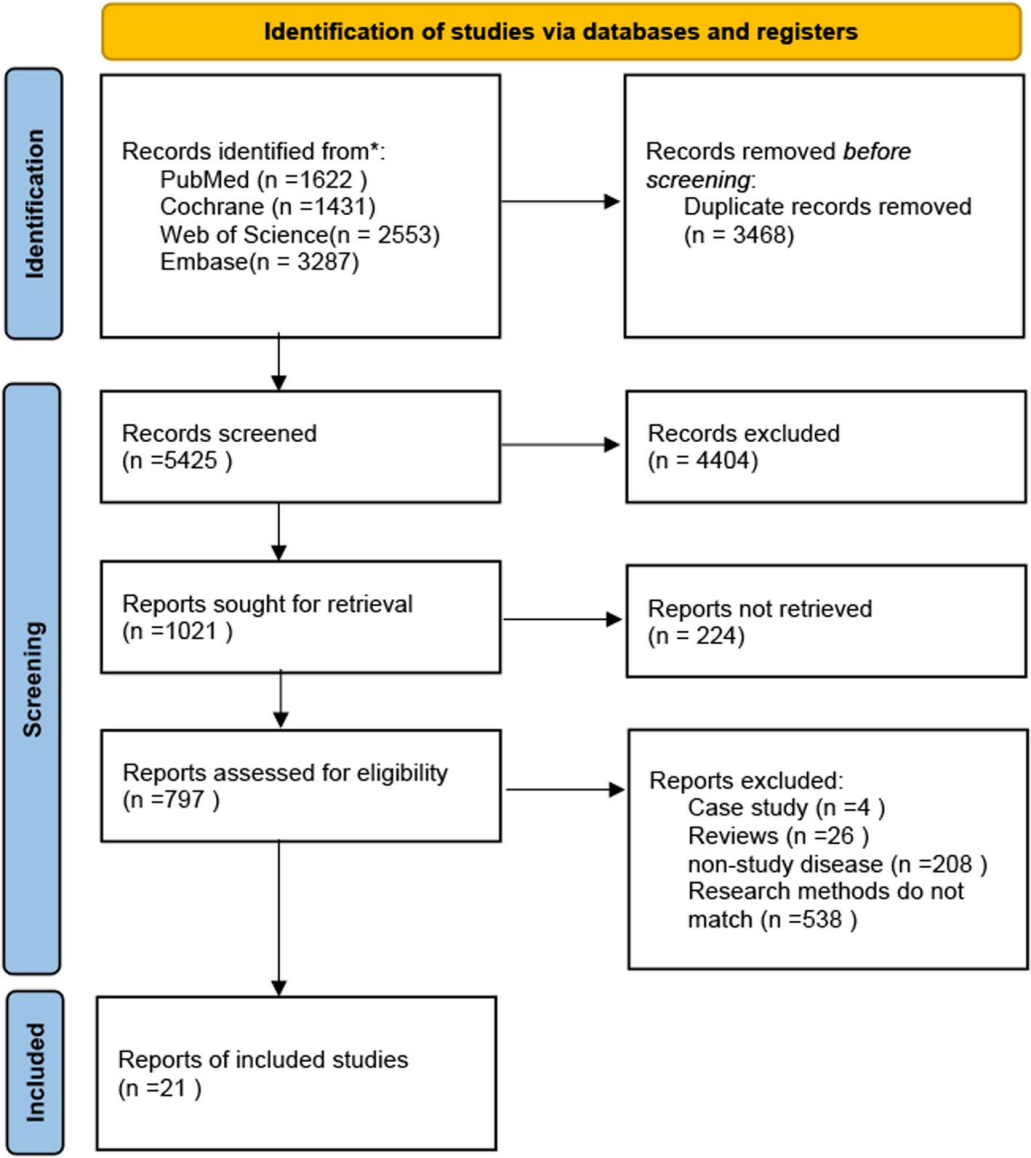
Flowchart of all studies identified, included and excluded

A total of 8893 articles were identified through database searches. After importing the retrieved literature into EndNote X9, 3468 duplicate articles were removed. Following the screening of titles and abstracts, 4404 irrelevant articles were excluded. Subsequently, 994 articles that did not meet the criteria were removed, resulting in the inclusion of 21 articles. The literature screening process and results are shown in Fig. 1.

### Basic characteristics of included studies

A total of 21 [14–16, 4, 17–33] articles involving 3030 study participants were included, comprising 1509 individuals in the experimental group and 1521 individuals in the control group. The experimental group received telerehabilitation, mainly including telemedicine video consultation, Virtual Autonomous Physiotherapist Agent, video-guided exercises, etc. While the control group received standard care. 6WMD, SGRQ and mMRC are the main outcome indicators used in this study to measure patient improvement. All RCT intervention-related literature was in English. The basic characteristics of the included studies are outlined in Table 1.

**Table 1:** Characteristics of included studies

Author, Year	Country	Type of work	Male/Female		Age		Sample Size (E/C)	Treatment		Disease	Follow Up time	Outcome indicators
			**Experiment group**	**Control group**	**Experiment group**	**Control group**		**Experiment group**	**Control group**			
Paolo Zanaboni 2022	Norway	RCT	23/17	43/37	64.9(7.1)	63.8(7.8)	40/80	Integrated intervention consisting of exercise training at home, telemonitoring, and self-management	1. Unsupervised Training 2. Control	COPD	2 years	6MWD; mMRC; CAT
Roberto Benzo 2022	Florida	RCT	87/101	76/111	69.3(9.5)	68.7(9.5)	188/187	Weekly HC calls and a remote monitoring system	Usual care	COPD	6 months	mMRC
Narelle S Cox 2021	Australia	RCT	30/38	36/31	68(9.0)	67.0(9.0)	68/67	Provided with telerehabilitaiton equipment ‘kit’	Centre-based pulmonary rehabilitation	COPD	1 year	6MWD; mMRC; HADS
Jose Cerdán-de-las-Heras 2021	Switzerland	RCT	16/11	15/12	67.4(10.2)	72.5(7.4)	27/27	Tele-Rehabilitation with Virtual Autonomous Physiotherapist Agent (VAPA), a Eurostars-funded platform built by a European collaboration	Standard Rehabilitation	COPD	6 months	6MWD; SGRQ
Lingling Wang 2021	China	RCT	14/16	15/15	55.9(7.2)	56.7(6.3)	30/30	12 Weeks of home-based PR and conventional drug treatment	Conventional drug treatment	Interstitial lung disease (ILD)	1 year	6MWD; mMRC; PF
Dr Aroub Lahham PhD (Physio) 2020	Australia	RCT	17/12	17/12	68.0(9.0)	67.0(10.0)	29/29	Eight weeks of home-based PR (one home visit and seven once-weekly telephone calls)	Standard care (weekly social telephone calls)	COPD	6 months	6MWD; mMRC
Henrik Hansen 2020	Denmark	RCT	32/35	28/39	68.4(8.7)	68.2(9.4)	67/67	Pulmonary tele-rehabilitation programme	Conventional pulmonary rehabilitation	COPD	22 weeks	6MWD; CAT; HADS
Nina Godtfredsen 2020	Denmark	RCT	30/37	30/37	68.3(9.0)	68.3(9.0)	67/67	On-line, supervised and home-based tele-rehabilitation (intervention group)	Standardised, outpatient pulmonary rehabilitation (control group)	COPD	1 year	6MWD; CAT; HADS
Yi Li 2018	China	RCT	71/11	55/14	65.1(8.7)	66.0(9.3)	82/69	PR maintenance group (PRMG)-home-visit and phone contact	Usual care group	COPD	1 year	PF
Kate Jolly 2018	Multicentre	RCT	183/106	183/105	70.7(8.8)	70.2(7.8)	289/288	Telephone health coaching delivered by a nurse with supporting written documents,a pedometer,and a self monitoring diary	Usual care	COPD	2 years	SGRQ; HADS
Elizabeth J Horton 2017	UK	RCT	93/52	94/48	68.0(9.0)	67.0(8.0)	145/142	Home-based (The SPACE for COPD Manual)	Centre-based	COPD	6 months	HADS
Maroula Vasilopoulou 2017	Greece	RCT	44/3	75/25	67.0(9.6)	65.4(7.7)	47/100	Home-based maintenance tele-rehabilitation programme	1. Hospital-based,outpatient,maintenance rehabilitation programme 2. Usual care treatment	COPD	1 year	6MWD; mMRC; CAT
LING LING Y. TSAI 2016	Sydney	RCT	12/7	6/11	73.0(8.0)	75.0(9.0)	19/17	Home-based telerehabilitation group that received exercise training three times a week for 8 weeks	Usual care without exercise training	COPD	8 weeks	6MWD; CAT; HADS
Anne E Holland 2016	Australia	RCT	48/32	51/35	69(13)	69(10)	80/86	A new home-based model including one home visit and seven once-weekly telephone calls from a physiotherapist	Standard outpatient centre-based model	COPD	1 year	6MWD; mMRC
Helen Laura Cameron Tucker 2016	Australia	RCT	16/19	13/17	68(9.9)	70(6.8)	35/30	Health-mentoring targeting home-based walking(tele-rehab)	Usual care	COPD	12 weeks	6MWD; CAT
Julia Billington 2014	United Kingdom	RCT	18/17	17/21	72.1(9.2)	72.0(11.0)	35/38	Nurse telephone support	Received standard care including a self-management plan	COPD	12 weeks	CAT
Juliana M. de Sousa Pinto 2014	Spain	RCT	22/1	17/1	68.9(9.2)	71.9(7.6)	23/18	The TG participated in a 12-week home-based PR program in addition to the standard medical therapy and an individual nursing counseling session regarding the use and handling of inhaler devices and nebulizer therapy	Standard medical therapy and the nursing counseling session	COPD	3 months	6MWD; SGRQ; mMRC
Eric Y. Wong 2013	Canada	RCT	60/51	27/30	69.5(9.6)	69.8(9.0)	111/57	1. Provided telephone support through a series of eight phone calls over a six month period from peer educators (PS group) 2. Provided telephone support through a series of eight phone calls over a six month period from respiratory therapist s(RSgroup)	Usual care	COPD	6 months	6MWD; SGRQ
Julia Walters 2013	Australian	RCT	49/41	47/45	68.2(7.9)	67.3(7.6)	90/92	Health mentor (HM) group received regular calls to manage illness issues and health behaviours from trained community health nurses using negotiated goal setting: problem solving, decisionmaking and action planning	Usual care (UC) group received GP care plus non-interventional brief phone calls	COPD	1 year	SGRQ; HADS
Lone Schou 2013	Denmark	RCT	10/12	8/14	68(12)	73(10)	22/22	Intervention group with home telemedicine equipment	Control group who had conventional hospital admission	COPD	3 months	SGRQ; HADS
Eui-Geum Oh 2003	South Korea	RCT	10/5	4/4	64.8(7.8)	66.8(2.3)	15/8	Home-based pulmonary rehabilitation program, composed of inspiratory muscle training, upper and lower extremity exercise, relaxation, and telephone visit	Control group (given educational advice)	COPD	8 weeks	6MWD; SGRQ; mMRC; CAT; HADS; PF

*RCT* Randomized controlled trial, *6MWD* 6-min walking distance, *Mmrc* modified Medical Research Council Dyspnea Scale, *CAT* COPD assessment test, *SGRQ* St. George’s Respiratory Questionnaire, *HADS* Hospital anxiety and depression scale, *PF* Pulmonary function, *COPD* chronic obstructive pulmonary disease

### Assessment of bias risk in included studies

**Fig. 2:**
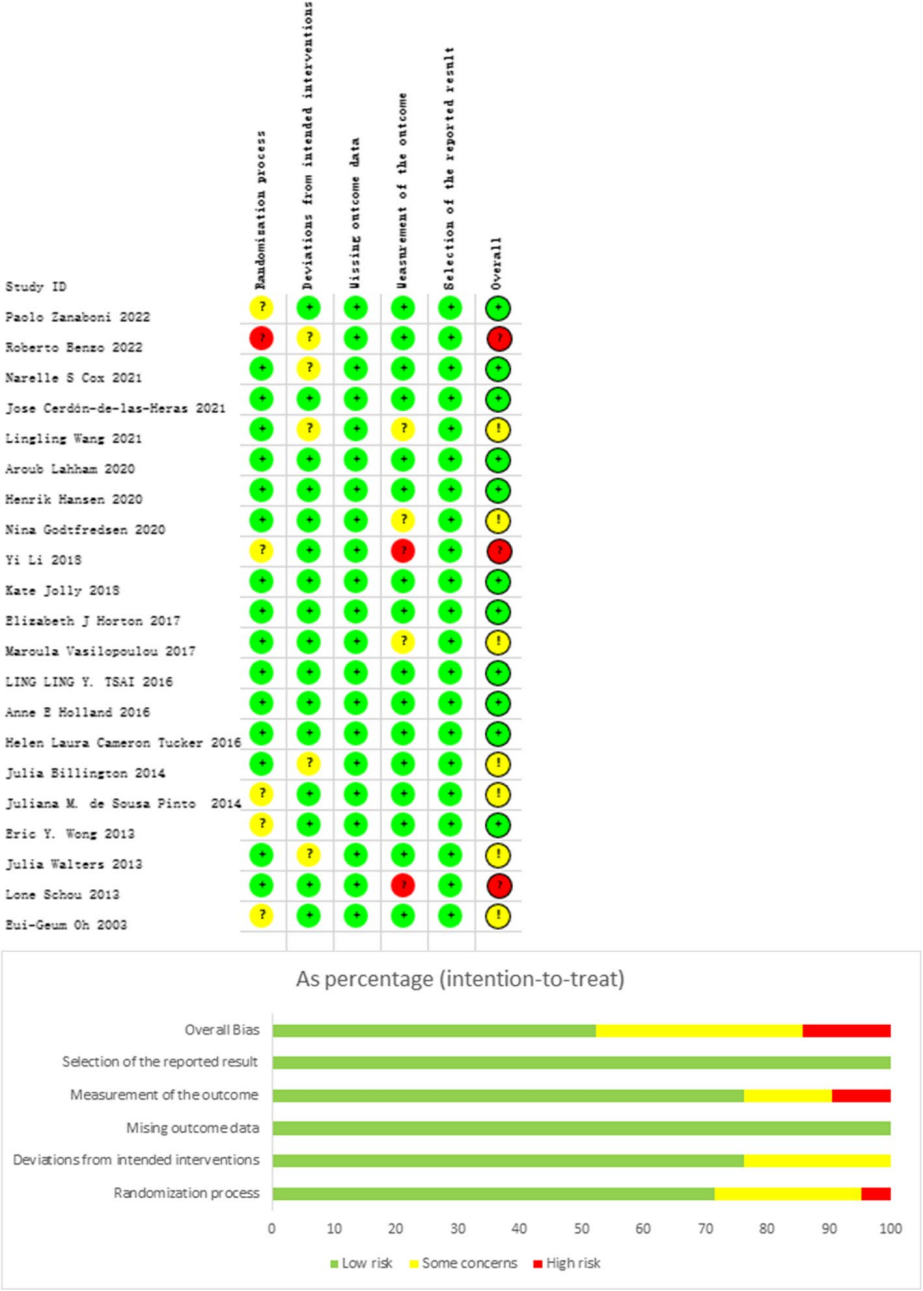
Risk of bias ratio plot

The quality of the included studies was assessed using the ROB2 tool recommended by Cochrane. Among the 21 trials, the majority of the literature described the randomization process, including whether blinding was utilized, such as through computer-generated random numbers or randomization tables. However, due to the nature of the intervention and certain outcomes (such as self-reported quality of life), there are indeed some biases that cannot be entirely avoided. To more accurately reflect the risk of bias, we considered the specific outcomes in our assessment and performed a detailed analysis for each included randomized controlled trial. Figure 2 presents the risk of bias summary for each trial.

### Meta-analysis results

#### Meta-analysis results of 6MWD

**Fig. 3:**
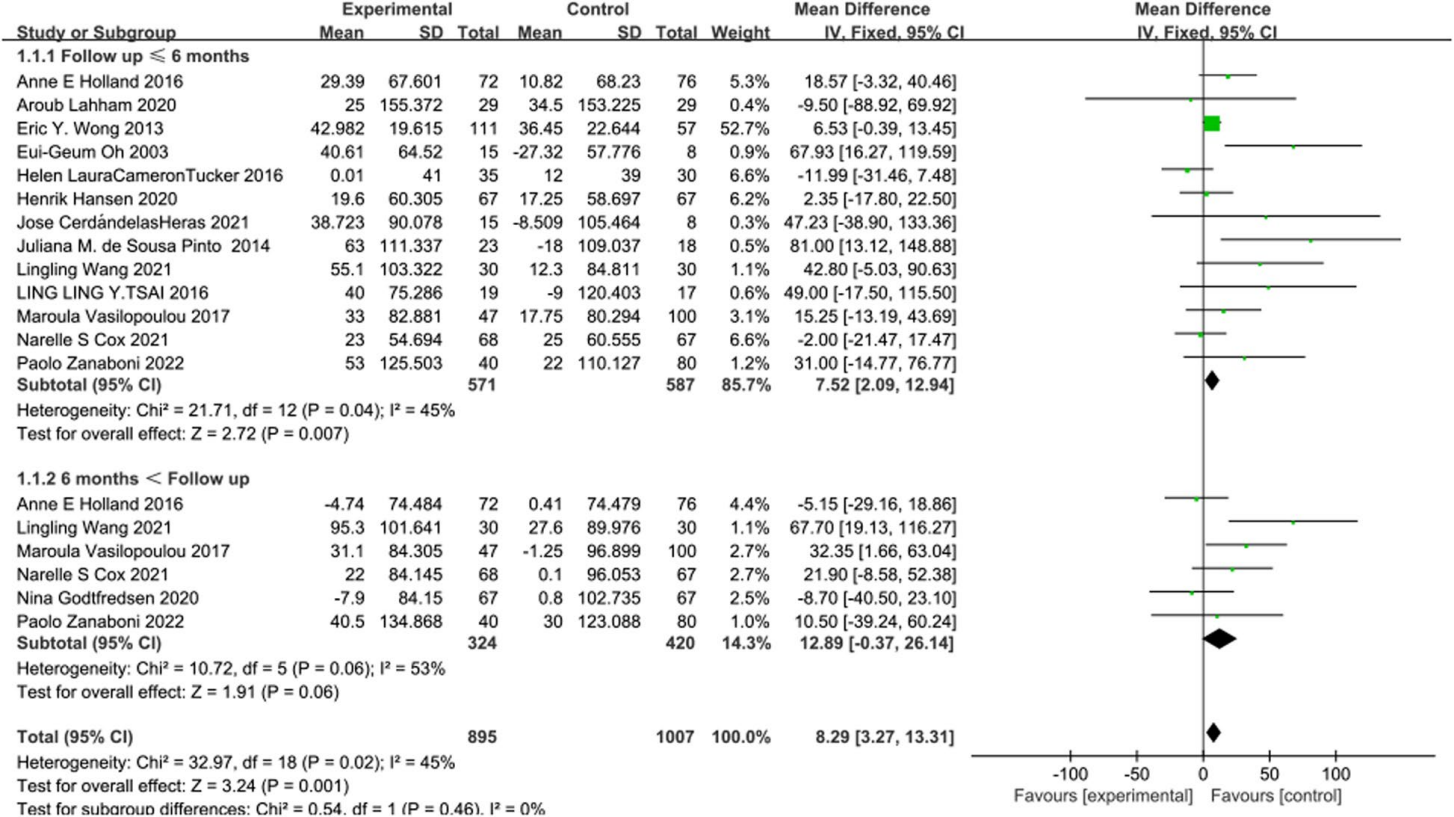
Forest plot of 6MWD

The intervention effect of telerehabilitation on CRD was reported in 14 [14–26, 4] studies, comprising 638 participants in the experimental group and 654 participants in the control group. Using a fixed-effects model (*I*^2^ = 45%, *P* = 0.02) for effect size pooling, the analysis revealed that compared to conventional rehabilitation in the control group, telerehabilitation in the experimental group demonstrated a significant improvement in outcomes at ≤ 6 months post-intervention [WMD = 7.52, 95%CI (2.09, 12.94)]( See Fig. 3). However, when > 6 months [WMD = 12.89, 95%CI (-0.37, 26.14)], there was no statistically significant difference between the experimental and control groups.

#### Meta-analysis results of mMRC

**Fig. 4:**
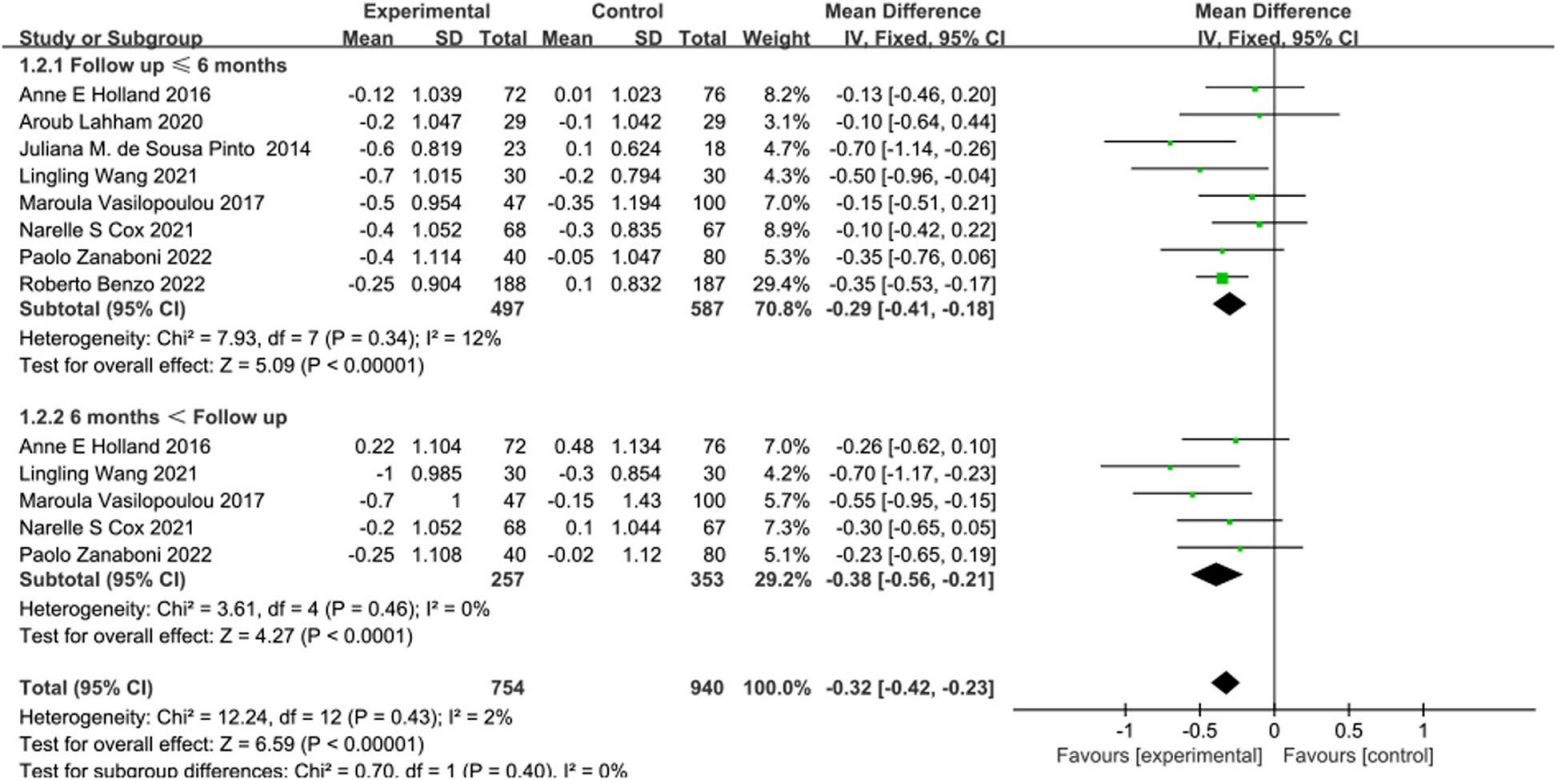
Forest plot of mMRC

The intervention effect of telerehabilitation on CRD was reported in 8 [14, 15, 21, 4, 23–25, 28] studies using mMRC, comprising 497 participants in the experimental group and 587 participants in the control group. Employing a fixed-effects model (*I*^2^ = 2%, *P* = 0.43) for effect size pooling, the analysis revealed that compared to conventional rehabilitation in the control group, telerehabilitation in the experimental group showed significant improvement in outcomes both at ≤ 6 months post-intervention [WMD = -0.29, 95%CI (-0.41, -0.18)] and > 6 months [WMD = -0.38, 95%CI (-0.56, -0.21)]. (shown in Fig. 4).

#### Meta-analysis results of SGRQ

**Fig. 5:**
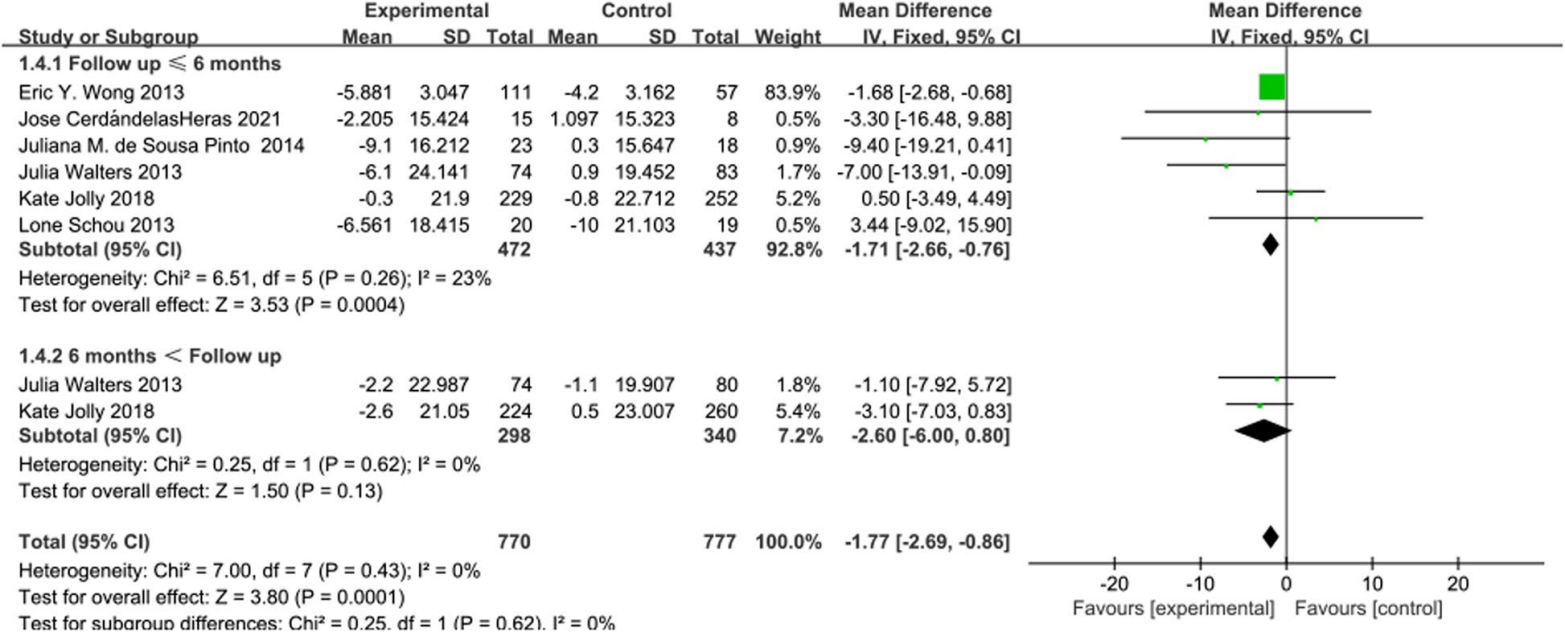
Forest plot of SGRQ (Activity score)

Group discussions were conducted on the SGRQ based on Activity score, impact score, and symptom score. In 6 [16, 20, 21, 30–32] studies reporting Activity score, telerehabilitation intervention effects on CRD were examined, with 472 participants in the intervention group and 445 participants in the control group. Using a fixed-effects model (*I*^2^ = 0%, *P* = 0.43) for effect size pooling, the analysis results revealed that compared to conventional rehabilitation in the control group, telerehabilitation in the intervention group demonstrated a significant improvement in outcomes at ≤ 6 months post-intervention [WMD = -1.71, 95%CI (-2.66, -0.76)] (See Fig. 5). However, when > 6 months [WMD = -2.60, 95%CI (-6.00, 0.80)], no statistically significant difference between the intervention and control groups was noticed.

**Fig. 6:**
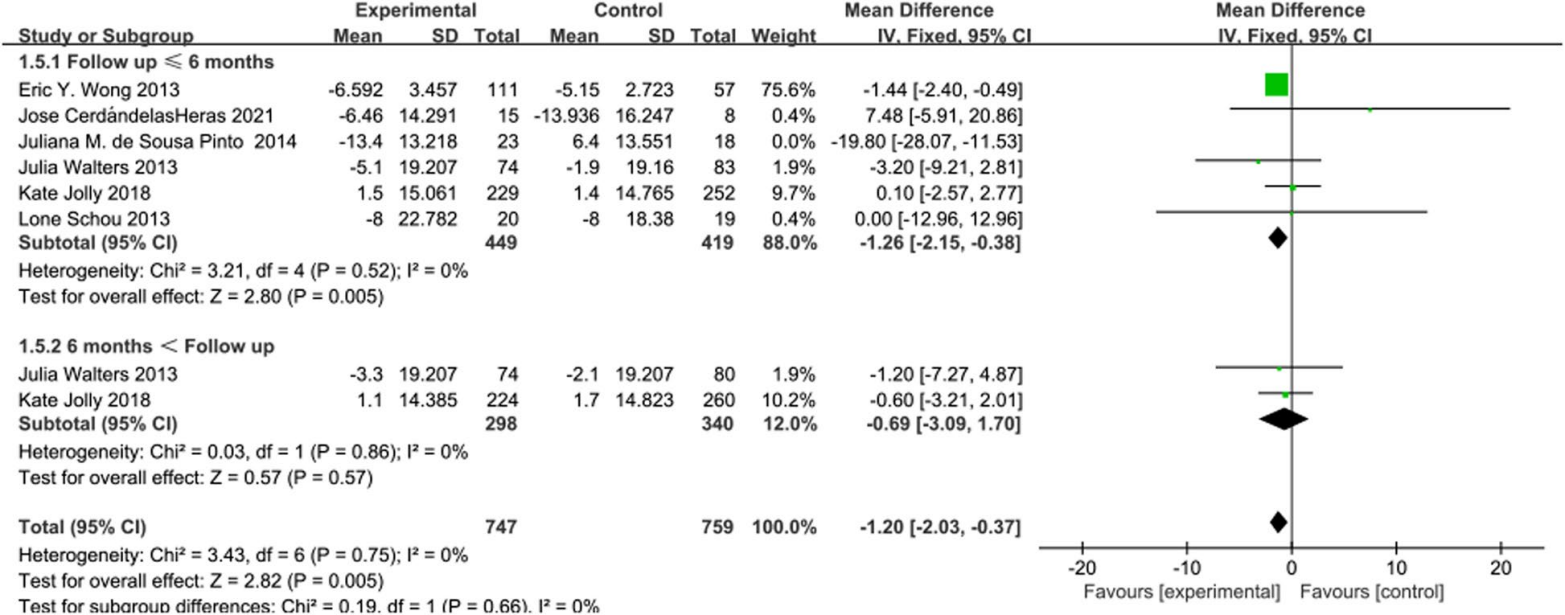
Forest plot of SGRQ (Impact score)

In 6 [16, 20, 21, 30–32]studies reporting Impact score, the intervention effects of telerehabilitation on CRD were examined, with 449 participants in the intervention group and 427 participants in the control group. Using a fixed-effects model (*I*^2^ = 0%, *P* = 0.75) for effect size pooling, the analysis results indicated that compared to conventional rehabilitation in the control group, telerehabilitation in the intervention group demonstrated a significant improvement in outcomes at ≤ 6 months post-intervention [WMD = -1.26, 95%CI (-2.15, -0.38)] (See Fig. 6). However, when > 6 months [WMD = -0.69, 95%CI (-3.09, 1.70)], there was no statistically significant difference between the intervention and control groups.

**Fig. 7:**
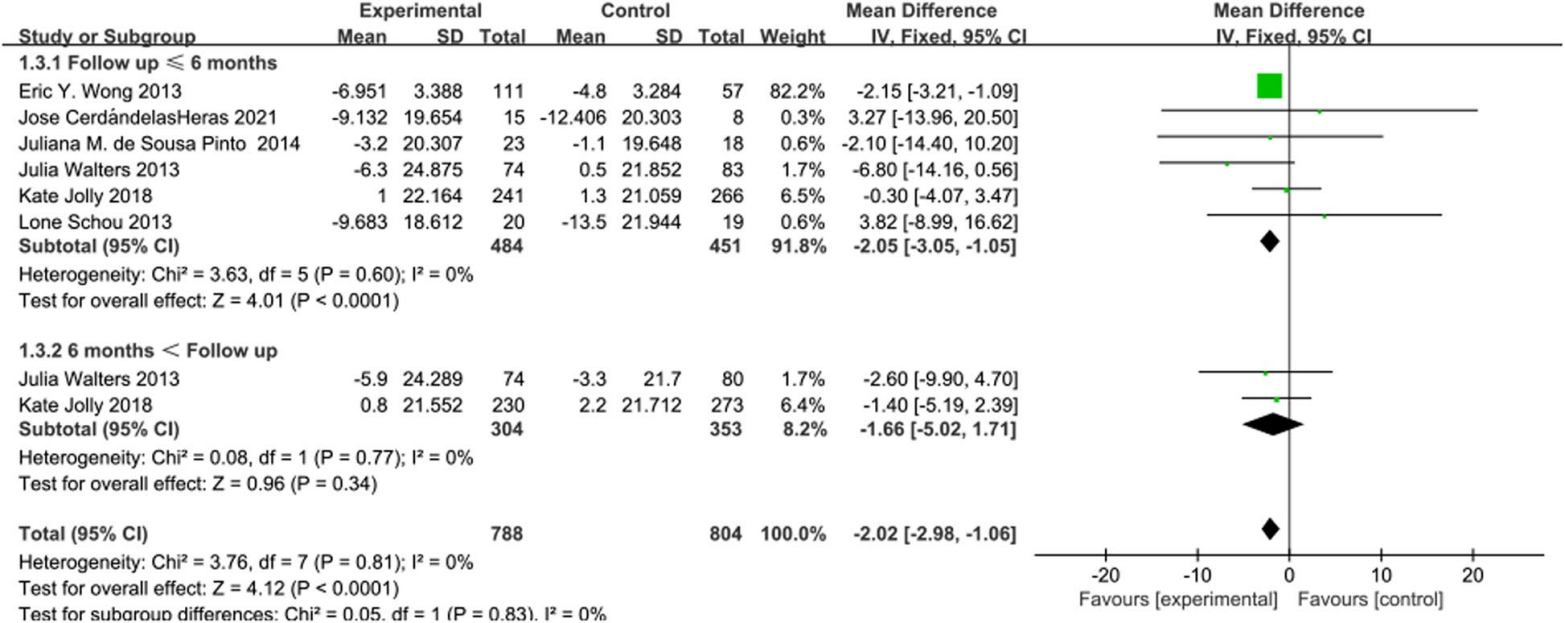
Forest plot of SGRQ (Symptom score)

In 6 [16, 20, 21, 30–32] studies reporting Symptom score, the intervention effects of telerehabilitation on CRD were examined, with 484 participants in the intervention group and 458 participants in the control group. Employing a fixed-effects model (*I*^2^ = 0%, *P* = 0.81) for effect size pooling, the analysis results demonstrated that compared to conventional rehabilitation in the control group, telerehabilitation in the intervention group exhibited a significant improvement in outcomes at ≤ 6 months post-intervention [WMD = -2.05, 95%CI (-3.05, -1.05)] (See Fig. 7). However, when > 6 months [WMD = -1.66, 95%CI (-5.02, 1.71)], there was no statistically significant difference between the intervention and control groups.

#### Meta-analysis results of CAT

**Fig. 8:**
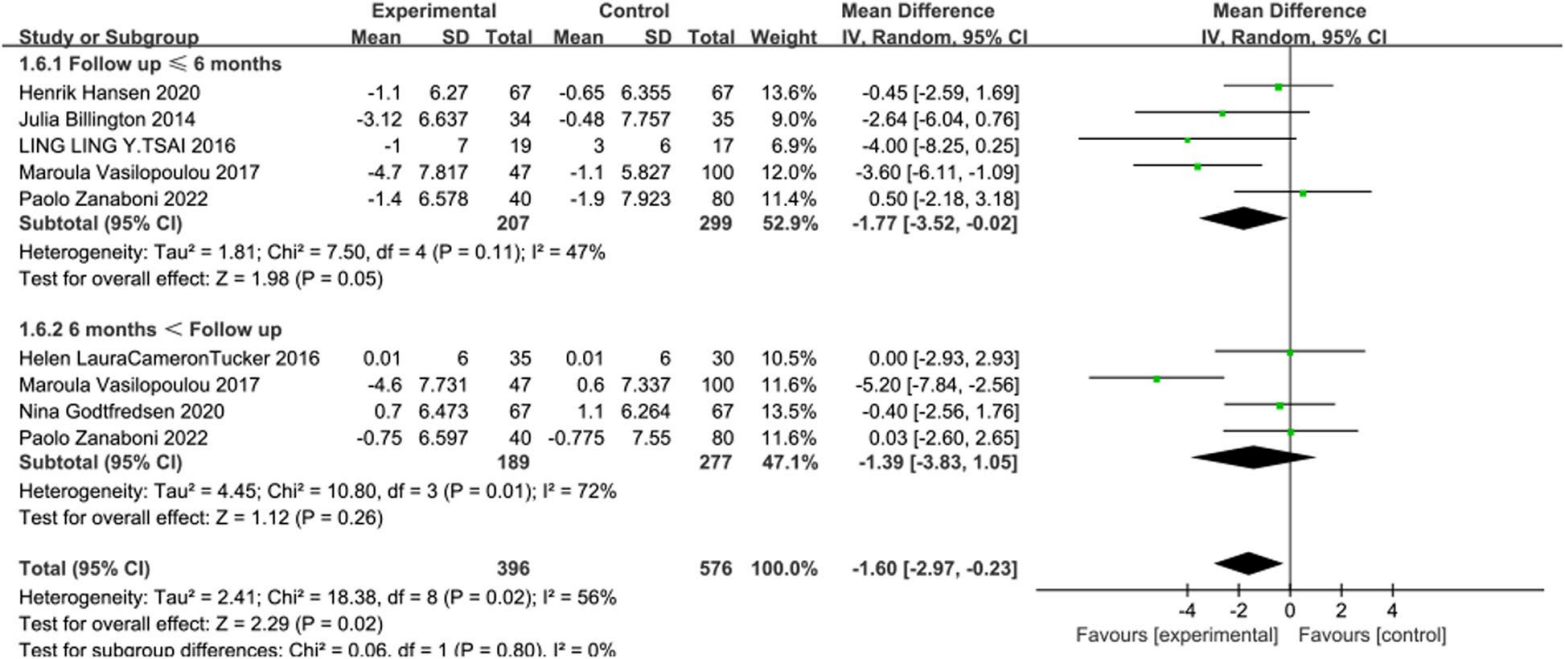
Forest plot of CAT

In 7 [18, 4, 19, 22, 24, 26, 27] studies reporting CAT, the intervention effects of telerehabilitation on CRD were examined, with 309 participants in the intervention group and 396 participants in the control group. Utilizing a random-effects model (*I*^2^ = 56%, *P* = 0.02) for effect size pooling, the analysis results indicated that compared to conventional rehabilitation in the control group, telerehabilitation in the intervention group exhibited a significant improvement in outcomes at ≤ 6 months post-intervention [WMD = -1.77, 95%CI (-3.52, -0.02)] (See Fig. 8). However, when > 6 months [WMD = -1.39, 95%CI (-3.83, 1.05)], there was no statistically significant difference between the intervention and control groups.

#### Meta-analysis results of pulmonary function

**Fig. 9:**
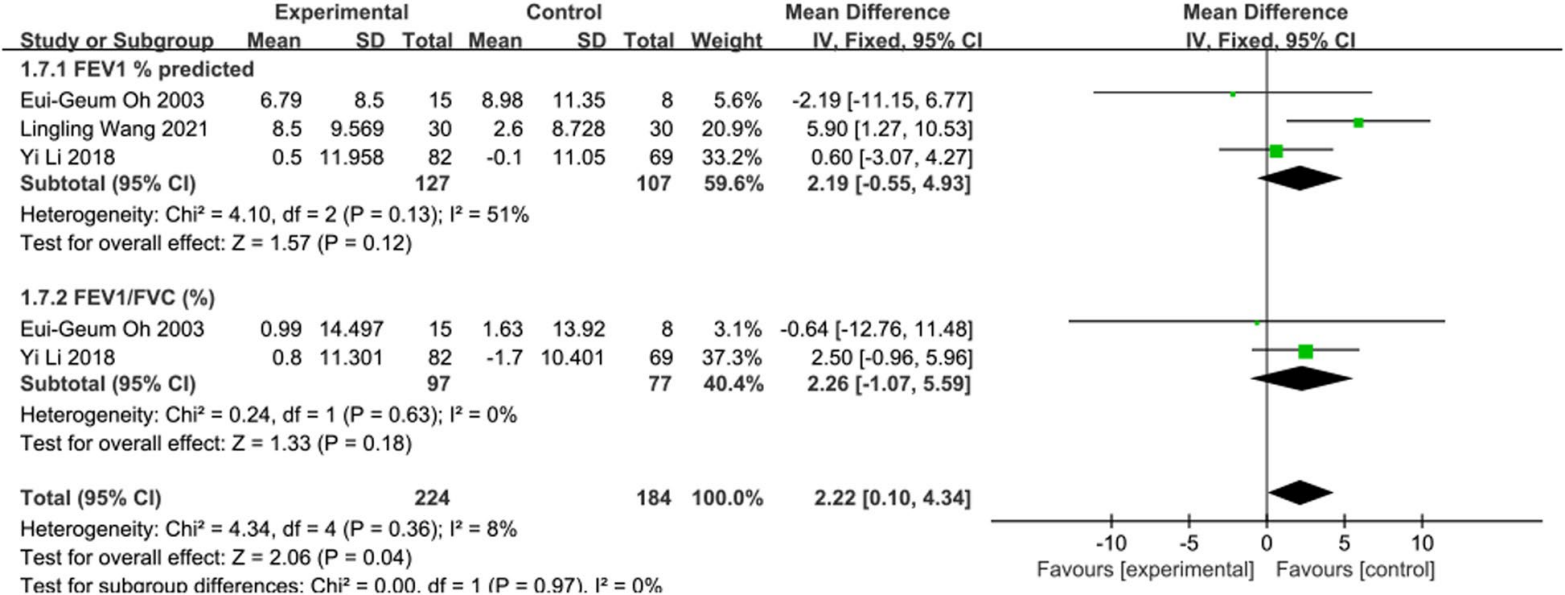
Forest plot of Pulmonary Function

Subgroup analysis of Pulmonary function was conducted based on FEV1% predicted and FEV1/FVC (%). In 3 [17, 23, 33] studies reporting FEV1% predicted, the intervention effects of telerehabilitation on CRD were examined, with 127 participants in the intervention group and 107 participants in the control group. Employing a fixed-effects model (*I*^2^ = 51%, *P* = 0.13) for effect size pooling, the analysis results showed that there was no statistically significant difference between the intervention and control groups in terms of FEV1% predicted [WMD = 2.19, 95%CI (-0.55, 4.93)] (See Fig. 9).

In 2 [17, 33] studies reporting FEV1/FVC (%), the intervention effects of telerehabilitation on CRD were examined, with 97 participants in the intervention group and 77 participants in the control group. Employing a fixed-effects model (*I*^2^ = 0%, *P* = 0.63) for effect size pooling, the analysis results indicated that there was no statistically significant difference between the intervention and control groups in terms of FEV1/FVC (%) [WMD = 2.26, 95%CI (-1.07, 5.59)] (See Fig. 9).

#### Meta-analysis results of HADS

**Fig. 10:**
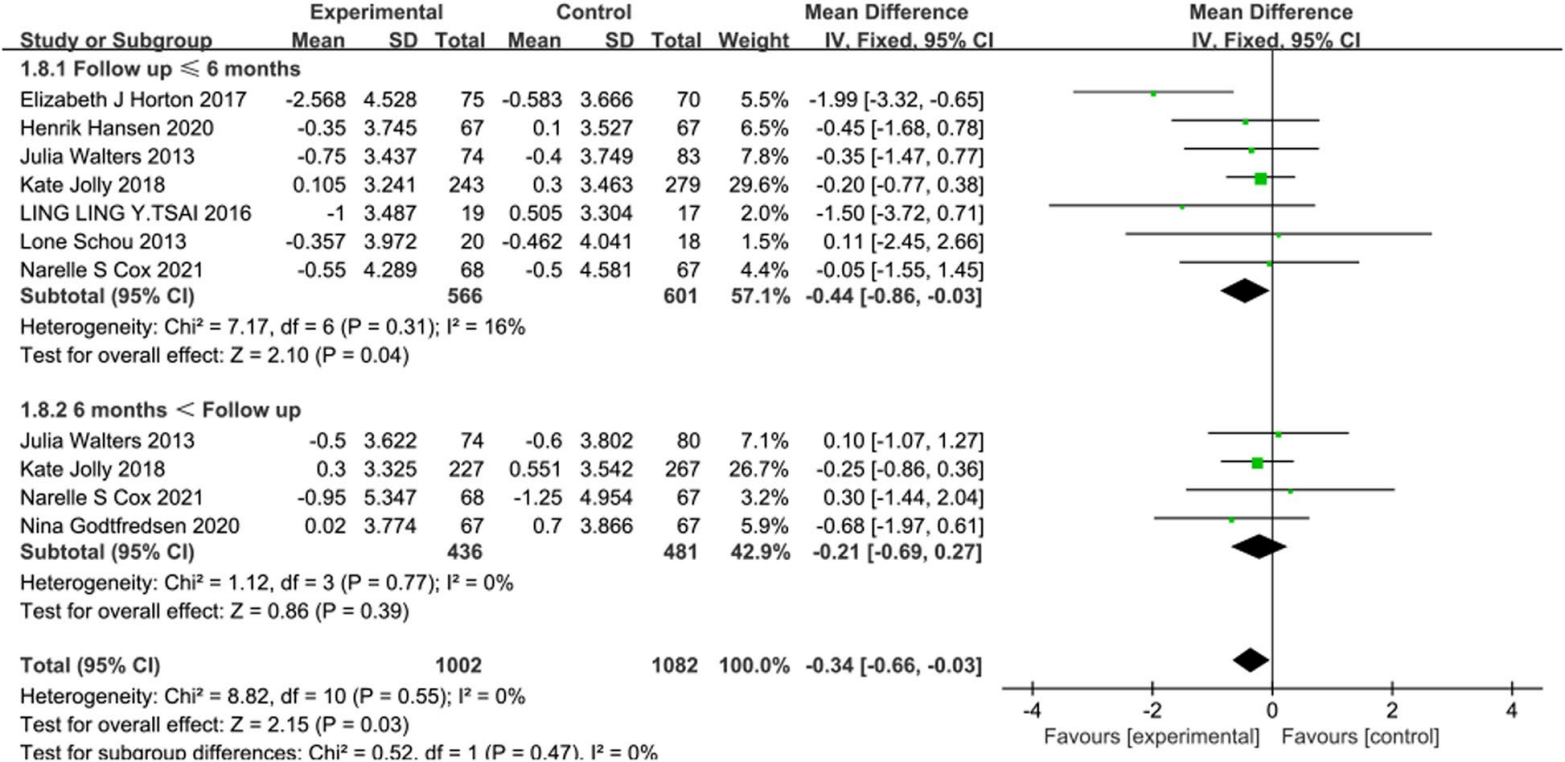
Forest plot of HADS

In 8 [19, 22, 25, 26, 29–32] studies reporting HADS, the intervention effects of telerehabilitation on CRD were examined, with 633 participants in the intervention group and 668 participants in the control group. Utilizing a fixed-effects model (*I*^2^ = 0%, *P* = 0.55) for effect size pooling, the analysis results indicated that compared to conventional rehabilitation in the control group, telerehabilitation in the intervention group exhibited a significant improvement in outcomes at ≤ 6 months post-intervention [WMD = -0.44, 95%CI (-0.86, -0.03)] (See Fig. 10). However, when > 6 months [WMD = -0.21, 95%CI (-0.69, 0.27)], there was no statistically significant difference between the intervention and control groups.

#### Publication bias and sensitivity analysis

Publication bias analysis was conducted for each included indicator using a funnel plot to visually display publication bias. Egger’s test was utilized to analyze the funnel plot, with a *p*-value > 0.05 indicating the absence of publication bias. Egger’s test revealed a *p*-value of 0.019 for the 6MWD indicator (Table 2), indicating the presence of publication bias among the studies. Therefore, for indicators exhibiting publication bias, a trim-and-fill method was employed for further analysis. After incorporating six additional studies into the model to achieve funnel plot symmetry, the combined effect size for the 6MWD indicator was 5.836, with a 95% confidence interval of (0.925, 10.746) (Table 3).

**Table 2:** Egger’s test results for publication bias

Outcome Indicator	Parameter	Effect Size	Standard Error	**95%CI**	*t*-value	*p*-value
6MWD	slope	-1.299	4.785	-11.395,8.797	-0.27	0.789
	bias	1.107	0.429	0.203,2.012	2.58	0.019
SGRQ -Activity score	slope	-1.462	0.646	-3.044,0.120	-2.26	0.064
	bias	-0.355	0.490	-1.554,0.845	-0.72	0.496
SGRQ -impact score	slope	-0.995	1.382	-4.832,2.842	-0.72	0.511
	bias	-0.649	1.257	-4.138,2.840	-0.53	0.633
SGRQ -symptom score	slope	-2.232	0.505	-3.466,-0.997	-4.42	0.004
	bias	0.230	0.364	-0.660,1.120	0.63	0.550
mMRC	slope	-0.247	0.161	-0.602,0.180	-1.53	0.154
	bias	-0.441	0.921	-2.467,1.586	-0.48	0.642
CAT	slope	2.085	3.694	-6.650,10.821	0.56	0.590
	bias	-2.673	2.730	-9.128,3.781	-0.98	0.360
HADS	slope	-0.082	0.351	-0. 877,0. 713	-0.23	0.820
	bias	-0.546	0.663	-2.047, 0.954	-0.82	0.431

**Table 3:** Trim-and-fill analysis results

Outcome indicators	Method	Phase	Pooled Est	95% CI	*z*	*p*	No. of studies
6MWD	Fixed	Before	8.288	(3.267,13.310)	3.235	0.001	19
		After	5.836	(0.925,10.746)	2.329	0.020	25

Sensitivity analysis was performed by individually excluding each study from the meta-analysis to assess the stability and reliability of the results. The sensitivity analysis results indicated that the meta-analysis results were stable and reliable.

## Discussion

Data indicates that approximately 3 million people die from COPD each year, with COPD projected to become the third leading cause of death worldwide by 2020 [34]. In 2013, the American Thoracic Society and the European Respiratory Society introduced a home-based pulmonary rehabilitation program aimed at providing pulmonary rehabilitation services for patients with COPD in the home environment. This pulmonary rehabilitation program involves comprehensive assessment of patients’ conditions and implementing integrated intervention measures based on personalized treatment [35]. However, due to issues such as resource shortages, high costs, and inconvenient transportation, out-of-hospital patients have lower compliance with pulmonary rehabilitation [36]. Remote home-based pulmonary rehabilitation, based on multimedia technology combined with computer and network technology, integrates with medical technology in large hospitals to provide remote online rehabilitation medical information and technical services. This form of rehabilitation service enables COPD patients to effectively integrate rehabilitation into their daily lives, while also reducing economic burdens to some extent, bringing certain benefits to patients [37]. It overcomes geographical limitations, to some extent addressing the shortage of medical resources in remote areas, further improving and enhancing the level of rehabilitation services in major cities, and greatly promoting the development of medical and healthcare industries. Currently, remote technology has been widely used to provide rehabilitation services for patients with COPD [22], asthma [38], heart failure [39], stroke [40], and other conditions.

A meta-analysis was performed, incorporating data 21 RCTs, to evaluate the effectiveness of remote pulmonary rehabilitation interventions for CRD. The study enrolled 1521 patients in the control group and 1509 patients in the intervention group. Primary outcome measures encompassed 6WMD, SGRQ, mMRC, CAT, HADS, and pulmonary function.

In the short term (≤ 6 months) observation, significant improvements were observed in 6WMD, mMRC, SGRQ, and CAT. The 6WMD reflected patients’ daily activity capacity, mMRC assessed the severity of dyspnea, SGRQ evaluated health status and quality of life, and CAT assessed disease severity and quality of life. However, it is important to note that the minimum clinically significant difference for the 6-min walking test is 30 m. Therefore, despite the statistical significance, the improvement observed does not reach the threshold for clinical relevance. The results of this study are consistent with previous research that has demonstrated the benefits of remote pulmonary rehabilitation in enhancing patients’ health status and quality of life. For example, Michaelchuk et al. (2022) [10] found similar improvements in CAT and mMRC following remote pulmonary rehabilitation in patients with COPD. These findings suggested that remote pulmonary rehabilitation interventions can substantially enhance patients’ activity capacity, alleviate dyspnea, and improve health status and quality of life in the short term. This improvement may be attributed to personalized rehabilitation plans provided by remote pulmonary rehabilitation and effective rehabilitation training facilitated by regular monitoring and guidance.

Notably, in long-term follow-up (> 6 months), while improvements in 6WMD, mMRC, SGRQ, and CAT still existed between the intervention and control groups, only the difference in mMRC was statistically significant. This may be due to increased loss to follow-up, reduced sample size, or decreased compliance of patients with remote pulmonary rehabilitation over the long term. Therefore, further long-term studies are needed to determine the long-term effects of remote pulmonary rehabilitation.

Furthermore, we conducted an evaluation using the widely adopted HADS, which is designed to assess anxiety and depression across various illnesses. The findings revealed significant improvements in HADS scores within ≤ 6 months post-intervention, indicating that telerehabilitation not only enhances the health status of patients but also ameliorates anxiety and depression among CRD patients, thereby enhancing their overall quality of life. However, over observation periods exceeding 6 months, there were no statistically significant differences observed between the intervention and control groups. This could be attributed to factors such as the chronic nature of airflow limitation in the disease, prolonged and slow disease progression, and decreased treatment adherence. It’s worth noting that due to the limited number of studies, we aggregated the anxiety and depression subscales for analysis. Future research is warranted to delve into separate analyses of anxiety and depression.

In terms of pulmonary function, this study conducted subgroup analysis based on FEV1% predicted and FEV1/FVC (%) and included a total of 5 studies reporting changes in pulmonary function outcomes. The results indicated no statistically significant differences between the intervention and control groups, which is consistent with the findings of Du et al. [41]. Given the possibility of insufficient sample sizes in the included studies, it is hoped that future research will involve long-term follow-up of these indicators to provide robust evidence for confirming the long-term effects of remote pulmonary rehabilitation.

Compared to previously published meta-analyses, this study’s strength lies in providing remote real-time interventions according to pulmonary rehabilitation measures and conducting statistical analyses of outcome indicators. It investigated the intervention effects of different time periods on CRD, thereby ensuring the research results are more rigorous and scientific. Additionally, it analyzed the anxiety and depression levels of patients, providing a comprehensive evaluation of the intervention effects of remote pulmonary rehabilitation on CRD. This offers more comprehensive evidence-based support for the real needs of CRD patients for remote pulmonary rehabilitation and provides targeted remote pulmonary rehabilitation services. The evidence from this study supports the effectiveness of remote pulmonary rehabilitation in improving alleviate dyspnea, and improve health status and quality of life for CRD patients, particularly in the short term. While the evidence is strong for COPD, more research is needed to determine the effectiveness of remote pulmonary rehabilitation in other respiratory diseases. To implement remote pulmonary rehabilitation at a national level in all pulmonary rehabilitation programs, it is essential to provide training for health personnel in the use of technological tools that enable them to deliver tailored interventions to respiratory patients. This training should encompass a range of professionals, including doctors, nurses, and physiotherapists, and should focus on familiarizing them with remote monitoring systems, online consultation platforms, and digital rehabilitation programs. Additionally, ongoing education and support should be provided to ensure that health personnel are proficient in utilizing these tools effectively and that they remain up-to-date with advancements in remote healthcare technology.

However, this study also has certain limitations. There were differences among the included study populations and baseline values, leading to higher heterogeneity in some positive results. Additionally, some studies did not describe allocation concealment and blinding methods, potentially introducing selection bias, implementation bias, and measurement bias. Moreover, the number of studies with long-term follow-up on efficacy was limited, possibly resulting in low test power. Furthermore, the economic benefits and costs associated with implementing telerehabilitation were not assessed in the included studies. It is hoped that future research will conduct more double-blind randomized controlled trials on the intervention effects of remote pulmonary rehabilitation in CRD, expand the sample size, extend the follow-up period, and observe outcome indicators comprehensively and with more standardized data, to provide more scientific evidence for the effectiveness and feasibility of remote pulmonary rehabilitation in CRD.

## Conclusion

The meta-analysis indicates that utilizing telerehabilitation therapy can improve respiratory function and mental health status in the short term, ultimately enhancing the quality of life for CRD patients. However, further evidence from more high-quality, large-sample randomized controlled trials is needed to establish the long-term effectiveness of this rehabilitation approach.

### Supplementary Information

Supplementary Material 1: Search Strategy.

Supplementary Material 2: Funnel plot and Sensitivity Analysis.

## Data Availability

All data generated or analysed during this study are included in this published article [and its supplementary information files].

## Abbreviations

CRD: Chronic respiratory disease
COPD: Chronic Obstructive Pulmonary Disease
CPH: China Pulmonary Health
TR: Telerehabilitation
PROSPERO: Preferred Reporting Items for Systematic Review and Meta-Analyses
6MWD: 6-Minute walk distance
SGRQ: St. George’s Respiratory Questionnaire
mMRC: Modified Medical Research Council Dyspnea Scale
CAT: COPD Assessment Test
HADS: Hospital Anxiety and Depression Scale
CI: Confidence interval
FEV1: Forced expiratory volume in the first one second
FVC: Forced vital capacity
RCT: Randomized controlled trial
