# Supplementary material for: The effects of telerehabilitation on physiological function and disease symptom for patients with chronic respiratory disease: a systematic review and meta-analysis
Source: BMC Pulm Med. 2024 Jun 28;24:305. doi: 10.1186/s12890-024-03104-8 (PMC11212271; doi:10.1186/s12890-024-03104-8)
Supplement: Supplementary file 1 — Supplementary Material 1: Search Strategy. [file 12890_2024_3104_MOESM1_ESM.docx]

**Supplementary Materials 1:** Search Strategy

**PubMed**

| Search number | Search Details | Results |
| --- | --- | --- |
| 1 | "pulmonary disease, chronic obstructive"[MeSH Terms] OR "pulmonary disease chronic obstructive"[Title/Abstract] OR "chronic obstructive lung disease"[Title/Abstract] OR "COAD"[Title/Abstract] OR "COPD"[Title/Abstract] OR "chronic obstructive airway disease"[Title/Abstract] OR "chronic obstructive pulmonary disease"[Title/Abstract] OR "airflow obstruction chronic"[Title/Abstract] OR "chronic airflow obstruction"[Title/Abstract] OR "chronic obstructive lung disease"[Title/Abstract] OR "chronic airflow obstruction"[Title/Abstract] OR "chronic airway obstruction"[Title/Abstract] OR "chronic obstructive bronchopulmonary disease"[Title/Abstract] OR "chronic obstructive lung disorder"[Title/Abstract] OR "chronic obstructive pulmonary disorder"[Title/Abstract] OR "chronic obstructive respiratory disease"[Title/Abstract] OR "chronic pulmonary obstructive disease"[Title/Abstract] OR "chronic pulmonary obstructive disorder"[Title/Abstract] OR (("Lung"[MeSH Terms] OR "Lung"[All Fields]) AND "chronic obstructive disease"[Title/Abstract]) OR "lung disease chronic obstructive"[Title/Abstract] OR "obstructive chronic lung disease"[Title/Abstract] OR "obstructive chronic pulmonary disease"[Title/Abstract] OR (("obstruct"[All Fields] OR "obstructed"[All Fields] OR "obstructing"[All Fields] OR "Obstruction"[All Fields] OR "obstructions"[All Fields] OR "Obstructive"[All Fields] OR "obstructs"[All Fields]) AND "lung disease chronic"[Title/Abstract]) OR "pulmonary disease chronic obstructive"[Title/Abstract] OR (("lung diseases"[MeSH Terms] OR ("Lung"[All Fields] AND "diseases"[All Fields]) OR "lung diseases"[All Fields] OR ("Pulmonary"[All Fields] AND "disorder"[All Fields]) OR "pulmonary disorder"[All Fields]) AND "chronic obstructive"[Title/Abstract]) | 109,340 |
| 2 | "Bronchiectasis"[MeSH Terms] OR "Bronchiectasis"[Title/Abstract] OR "Bronchiectases"[Title/Abstract] OR "Bronchiectasis"[Title/Abstract] OR "bronchiectasia"[Title/Abstract] OR "bronchoectasia"[Title/Abstract] | 16,107 |
| 3 | "lung diseases, interstitial"[MeSH Terms] OR "lung diseases interstitial"[Title/Abstract] OR "diffuse parenchymal lung disease"[Title/Abstract] OR "interstitial lung disease"[Title/Abstract] OR "lung disease interstitial"[Title/Abstract] OR "pneumonia interstitial"[Title/Abstract] OR "interstitial pneumonia"[Title/Abstract] OR "pneumonias interstitial"[Title/Abstract] OR "pneumonitis interstitial"[Title/Abstract] OR "interstitial pneumonitides"[Title/Abstract] OR "interstitial pneumonitis"[Title/Abstract] OR (("Pneumonia"[MeSH Terms] OR "Pneumonia"[All Fields] OR "Pneumonitides"[All Fields]) AND "Interstitial"[Title/Abstract]) OR "interstitial lung disease"[Title/Abstract] OR "diffuse interstitial pneumopathy"[Title/Abstract] OR ((("diffusable"[All Fields] OR "diffusant"[All Fields] OR "diffusants"[All Fields] OR "Diffuse"[All Fields] OR "diffusely"[All Fields] OR "diffuses"[All Fields] OR "diffusibility"[All Fields] OR "diffusible"[All Fields] OR "diffusion"[MeSH Terms] OR "diffusion"[All Fields] OR "diffused"[All Fields] OR "diffusing"[All Fields] OR "diffusions"[All Fields] OR "diffusive"[All Fields] OR "diffusively"[All Fields] OR "diffusivities"[All Fields] OR "diffusivity"[All Fields]) AND ("parenchyma"[All Fields] OR "parenchymas"[All Fields])) AND "lung disease"[Title/Abstract]) OR (("diffusable"[All Fields] OR "diffusant"[All Fields] OR "diffusants"[All Fields] OR "Diffuse"[All Fields] OR "diffusely"[All Fields] OR "diffuses"[All Fields] OR "diffusibility"[All Fields] OR "diffusible"[All Fields] OR "diffusion"[MeSH Terms] OR "diffusion"[All Fields] OR "diffused"[All Fields] OR "diffusing"[All Fields] OR "diffusions"[All Fields] OR "diffusive"[All Fields] OR "diffusively"[All Fields] OR "diffusivities"[All Fields] OR "diffusivity"[All Fields]) AND "parenchymal pulmonary disease"[Title/Abstract]) OR ((("diffusable"[All Fields] OR "diffusant"[All Fields] OR "diffusants"[All Fields] OR "Diffuse"[All Fields] OR "diffusely"[All Fields] OR "diffuses"[All Fields] OR "diffusibility"[All Fields] OR "diffusible"[All Fields] OR "diffusion"[MeSH Terms] OR "diffusion"[All Fields] OR "diffused"[All Fields] OR "diffusing"[All Fields] OR "diffusions"[All Fields] OR "diffusive"[All Fields] OR "diffusively"[All Fields] OR "diffusivities"[All Fields] OR "diffusivity"[All Fields]) AND ("parenchym"[All Fields] OR "Parenchymal"[All Fields] OR "parenchyme"[All Fields])) AND "pulmonary disorder"[Title/Abstract]) OR "interstitial lung disorder"[Title/Abstract] OR "interstitial pneumopathy"[Title/Abstract] OR "interstitial pulmonary disease"[Title/Abstract] OR (("Interstitial"[All Fields] OR "interstitials"[All Fields]) AND "pulmonary disorder"[Title/Abstract]) OR (("lung diseases"[MeSH Terms] OR ("Lung"[All Fields] AND "Diseases"[All Fields]) OR "lung diseases"[All Fields] OR "pneumopathy"[All Fields] OR "pneumopathies"[All Fields]) AND "Interstitial"[Title/Abstract]) OR "chronic respiratory diseases"[Title/Abstract] | 106,099 |
| 4 | #1 OR #2 OR #3 | 222,986 |
| 5 | "Telerehabilitation"[MeSH Terms] OR "Telerehabilitation"[Title/Abstract] OR "tele rehabilitation"[Title/Abstract] OR "remote rehabilitation"[Title/Abstract] OR "rehabilitation remote"[Title/Abstract] OR "virtual rehabilitation"[Title/Abstract] OR "rehabilitation virtual"[Title/Abstract] OR "Telerehabilitation"[Title/Abstract] OR "e-rehabilitation"[Title/Abstract] | 2,894 |
| 6 | "Telenursing"[MeSH Terms] OR "Telenursing"[Title/Abstract] OR "Telenursing"[Title/Abstract] OR "virtual nursing"[Title/Abstract] | 553 |
| 7 | "Telemedicine"[MeSH Terms] OR "Telemedicine"[Title/Abstract] OR "tele referral"[Title/Abstract] OR "virtual medicine"[Title/Abstract] OR "medicine virtual"[Title/Abstract] OR "tele intensive care"[Title/Abstract] OR "tele icu"[Title/Abstract] OR "mobile health"[Title/Abstract] OR "health mobile"[Title/Abstract] OR "mHealth"[Title/Abstract] OR "Telehealth"[Title/Abstract] OR "EHealth"[Title/Abstract] | 75,165 |
| 8 | "virtual reality"[MeSH Terms] OR "virtual reality"[Title/Abstract] OR "reality virtual"[Title/Abstract] OR "educational virtual realities"[Title/Abstract] OR (("Realities"[All Fields] OR "Reality"[All Fields]) AND "educational virtual"[Title/Abstract]) OR ((("Virtual"[All Fields] OR "virtuality"[All Fields] OR "virtualization"[All Fields] OR "virtualized"[All Fields] OR "virtualizing"[All Fields] OR "virtuals"[All Fields]) AND ("Realities"[All Fields] OR "Reality"[All Fields])) AND "Educational"[Title/Abstract]) OR (("instruct"[All Fields] OR "instructed"[All Fields] OR "instructing"[All Fields] OR "Instructional"[All Fields] OR "instructions"[All Fields] OR "instructive"[All Fields] OR "instructively"[All Fields] OR "instructiveness"[All Fields] OR "instructs"[All Fields] OR "teaching"[MeSH Terms] OR "teaching"[All Fields] OR "instruction"[All Fields]) AND "virtual realities"[Title/Abstract]) OR (("Realities"[All Fields] OR "Reality"[All Fields]) AND "instructional virtual"[Title/Abstract]) OR (("Realities"[All Fields] OR "Reality"[All Fields]) AND "instructional virtual"[Title/Abstract]) OR ((("Virtual"[All Fields] OR "virtuality"[All Fields] OR "virtualization"[All Fields] OR "virtualized"[All Fields] OR "virtualizing"[All Fields] OR "virtuals"[All Fields]) AND ("Realities"[All Fields] OR "Reality"[All Fields])) AND "Instructional"[Title/Abstract]) OR "virtual reality"[Title/Abstract] | 19,313 |
| 9 | "Home-based"[Title/Abstract] OR "Videogame"[Title/Abstract] OR "computergame"[Title/Abstract] OR "television game"[Title/Abstract] OR "tv games"[Title/Abstract] OR "computerized treatment"[Title/Abstract] OR "computerized training"[Title/Abstract] OR "computer-assisted"[Title/Abstract] | 47,299 |
| 10 | #5 OR #6 OR #7 OR #8 OR #9 | 140,061 |
| 11 | #4 AND #10 | 1,622 |

**Cochrane Library**

Search Name:

Date Run: 14/11/2023 16:57:39

Comment:

ID Search Hits

#1 MeSH descriptor: [Pulmonary Disease, Chronic Obstructive] explode all trees 7317

#2 (Pulmonary Disease, Chronic Obstructive):ti,ab,kw OR (Chronic Obstructive Lung Disease):ti,ab,kw OR (COAD):ti,ab,kw OR (COPD):ti,ab,kw OR (Chronic Obstructive Airway Disease):ti,ab,kw 24328

#3 (Chronic Obstructive Pulmonary Disease):ti,ab,kw OR (Airflow Obstruction, Chronic):ti,ab,kw OR (Chronic Airflow Obstruction):ti,ab,kw OR (chronic obstructive lung disease):ti,ab,kw OR (chronic airflow obstruction):ti,ab,kw 19040

#4 (chronic airway obstruction):ti,ab,kw OR (chronic obstructive bronchopulmonary disease):ti,ab,kw OR (chronic obstructive lung disorder):ti,ab,kw OR (chronic obstructive pulmonary disorder):ti,ab,kw OR (chronic obstructive respiratory disease):ti,ab,kw 8397

#5 (chronic pulmonary obstructive disease):ti,ab,kw OR (chronic pulmonary obstructive disorder):ti,ab,kw OR (lung chronic obstructive disease):ti,ab,kw OR (lung disease, chronic obstructive):ti,ab,kw OR (obstructive chronic lung disease):ti,ab,kw 18862

#6 (obstructive chronic pulmonary disease):ti,ab,kw OR (obstructive lung disease, chronic):ti,ab,kw OR (pulmonary disease, chronic obstructive):ti,ab,kw OR (pulmonary disorder, chronic obstructive):ti,ab,kw OR (chronic respiratory diseases):ti,ab,kw 20752

#7 MeSH descriptor: [Bronchiectasis] explode all trees 547

#8 (Bronchiectasis):ti,ab,kw OR (Bronchiectases):ti,ab,kw OR (Bronchiectasis):ti,ab,kw OR (bronchiectasia):ti,ab,kw OR (bronchoectasia):ti,ab,kw 1503

#9 MeSH descriptor: [Lung Diseases, Interstitial] explode all trees 1594

#10 (Lung Diseases, Interstitial):ti,ab,kw OR (Diffuse Parenchymal Lung Disease):ti,ab,kw OR (Interstitial Lung Disease):ti,ab,kw OR (Lung Disease, Interstitial):ti,ab,kw OR (Pneumonia, Interstitial):ti,ab,kw 2337

#11 (Interstitial Pneumonia):ti,ab,kw OR (Pneumonias, Interstitial):ti,ab,kw OR (Pneumonitis, Interstitial):ti,ab,kw OR (Interstitial Pneumonitides):ti,ab,kw OR (Interstitial Pneumonitis):ti,ab,kw 1044

#12 (Pneumonitides, Interstitial):ti,ab,kw OR (interstitial lung disease):ti,ab,kw OR (diffuse interstitial pneumopathy):ti,ab,kw OR (diffuse parenchyma lung disease):ti,ab,kw OR (diffuse parenchymal pulmonary disease):ti,ab,kw 1921

#13 (diffuse parenchymal pulmonary disorder):ti,ab,kw OR (interstitial lung disorder):ti,ab,kw OR (interstitial pneumopathy):ti,ab,kw OR (interstitial pulmonary disease):ti,ab,kw OR (interstitial pulmonary disorder):ti,ab,kw 1113

#14 (pneumopathy, interstitial):ti,ab,kw 3

#15 #1or#2or#3or#4or#5or#6or#7or#8or#9or#10or#11or#12or#13or#14 31017

#16 MeSH descriptor: [Telerehabilitation] explode all trees 287

#17 (Telerehabilitation):ti,ab,kw OR (Tele rehabilitation):ti,ab,kw OR (Remote Rehabilitation):ti,ab,kw OR (Rehabilitation, Remote):ti,ab,kw OR (Virtual Rehabilitation):ti,ab,kw 3600

#18 (Rehabilitation, Virtual):ti,ab,kw OR (Telerehabilitation):ti,ab,kw OR (e-rehabilitation):ti,ab,kw 3040

#19 MeSH descriptor: [Telenursing] explode all trees 47

#20 (Telenursing):ti,ab,kw OR (Telenursing):ti,ab,kw OR (virtual nursing):ti,ab,kw 640

#21 MeSH descriptor: [Telemedicine] explode all trees 4313

#22 (Telemedicine):ti,ab,kw OR (Tele Referral):ti,ab,kw OR (Virtual Medicine):ti,ab,kw OR (Medicine, Virtual):ti,ab,kw OR (Tele Intensive Care):ti,ab,kw 6376

#23 (Tele ICU):ti,ab,kw OR (Mobile Health):ti,ab,kw OR (Health, Mobile):ti,ab,kw OR (mHealth):ti,ab,kw OR (Telehealth):ti,ab,kw 12871

#24 (EHealth):ti,ab,kw OR (telemedicine):ti,ab,kw 7017

#25 MeSH descriptor: [Virtual Reality] explode all trees 1022

#26 (Virtual Reality):ti,ab,kw OR (Reality, Virtual):ti,ab,kw OR (Educational Virtual Realities):ti,ab,kw OR (Reality, Educational Virtual):ti,ab,kw OR (Virtual Realities, Educational):ti,ab,kw 6268

#27 (Instructional Virtual Realities):ti,ab,kw OR (Realities, Instructional Virtual):ti,ab,kw OR (Reality, Instructional Virtual):ti,ab,kw OR (Virtual Realities, Instructional):ti,ab,kw OR (virtual reality):ti,ab,kw 6268

#28 (Videogame):ti,ab,kw OR (computergame):ti,ab,kw OR (television game):ti,ab,kw OR (TV games):ti,ab,kw OR (Home-based):ti,ab,kw 9535

#29 (computerized treatment):ti,ab,kw OR (computerized training):ti,ab,kw OR (computer-assisted):ti,ab,kw 25558

#30 #16or#17or#18or#19or#20or#21or#22or#23or#24or#25or#26or#27or#28or#29 59179

#31 #15and#30 1450

**Embase**

Session Results

.......................................................

No. Query Results Results Date

#12. #4 AND #11 3,287 14 Nov 2023

#11. #5 OR #6 OR #7 OR #8 OR #9 OR #10 203,141 14 Nov 2023

#10. 'home based':ti,ab,kw OR 'computerized 60,665 14 Nov 2023

treatment':ti,ab,kw OR 'computerized

training':ti,ab,kw OR 'computer

assisted':ti,ab,kw

#9. 'videogame'/exp OR videogame:ti,ab,kw OR 7,232 14 Nov 2023

computergame:ti,ab,kw OR 'television

game':ti,ab,kw OR 'tv games':ti,ab,kw

#8. 'virtual reality'/exp OR 'virtual 33,278 14 Nov 2023

reality':ti,ab,kw OR 'reality, virtual':ti,ab,kw

OR 'educational virtual realities':ti,ab,kw OR

'reality, educational virtual':ti,ab,kw OR

'virtual realities, educational':ti,ab,kw OR

'instructional virtual realities':ti,ab,kw OR

'realities, instructional virtual':ti,ab,kw OR

'reality, instructional virtual':ti,ab,kw OR

'virtual realities, instructional':ti,ab,kw

#7. 'telemedicine'/exp OR telemedicine:ti,ab,kw OR 106,726 14 Nov 2023

'tele referral':ti,ab,kw OR 'virtual

medicine':ti,ab,kw OR 'medicine,

virtual':ti,ab,kw OR 'tele intensive

care':ti,ab,kw OR 'tele icu':ti,ab,kw OR 'mobile

health':ti,ab,kw OR 'health, mobile':ti,ab,kw OR

mhealth:ti,ab,kw OR telehealth:ti,ab,kw OR

ehealth:ti,ab,kw

#6. 'telenursing'/exp OR 'virtual nursing':ti,ab,kw 624 14 Nov 2023

OR telenursing:ti,ab,kw

#5. 'telerehabilitation'/exp OR 'e 3,852 14 Nov 2023

rehabilitation':ti,ab,kw OR

telerehabilitation:ti,ab,kw OR 'tele

rehabilitation':ti,ab,kw OR 'remote

rehabilitation':ti,ab,kw OR 'rehabilitation,

remote':ti,ab,kw OR 'virtual

rehabilitation':ti,ab,kw OR 'rehabilitation,

virtual':ti,ab,kw

#4. #1 OR #2 OR #3 377,169 14 Nov 2023

#3. 'interstitial lung disease'/exp OR 'diffuse 150,573 14 Nov 2023

interstitial pneumopathy':ti,ab,kw OR 'diffuse

parenchyma lung disease':ti,ab,kw OR 'diffuse

parenchymal pulmonary disease':ti,ab,kw OR

'diffuse parenchymal pulmonary disorder':ti,ab,kw

OR 'interstitial lung disorder':ti,ab,kw OR

'interstitial pneumopathy':ti,ab,kw OR

'interstitial pulmonary disease':ti,ab,kw OR

'interstitial pulmonary disorder':ti,ab,kw OR

'pneumopathy, interstitial':ti,ab,kw OR 'lung

diseases, interstitial':ti,ab,kw OR 'diffuse

parenchymal lung disease':ti,ab,kw OR

'interstitial lung disease':ti,ab,kw OR 'lung

disease, interstitial':ti,ab,kw OR 'pneumonia,

interstitial':ti,ab,kw OR 'interstitial

pneumonia':ti,ab,kw OR 'pneumonias,

interstitial':ti,ab,kw OR 'pneumonitis,

interstitial':ti,ab,kw OR 'interstitial

pneumonitides':ti,ab,kw OR 'interstitial

pneumonitis':ti,ab,kw OR 'pneumonitides,

interstitial':ti,ab,kw

#2. 'bronchiectasis'/exp OR bronchiectasia:ti,ab,kw 33,749 14 Nov 2023

OR bronchoectasia:ti,ab,kw OR

bronchiectasis:ti,ab,kw OR

bronchiectases:ti,ab,kw

#1. 'chronic obstructive lung disease'/exp OR 211,534 14 Nov 2023

'chronic airway obstruction':ti,ab,kw OR 'chronic

obstructive bronchopulmonary disease':ti,ab,kw OR

'chronic obstructive lung disorder':ti,ab,kw OR

'chronic obstructive pulmonary disorder':ti,ab,kw

OR 'chronic obstructive respiratory

disease':ti,ab,kw OR 'chronic pulmonary

obstructive disease':ti,ab,kw OR 'chronic

pulmonary obstructive disorder':ti,ab,kw OR 'lung

disease, chronic obstructive':ti,ab,kw OR

'obstructive chronic lung disease':ti,ab,kw OR

'obstructive chronic pulmonary disease':ti,ab,kw

OR 'obstructive lung disease, chronic':ti,ab,kw

OR 'pulmonary disorder, chronic

obstructive':ti,ab,kw OR 'pulmonary disease,

chronic obstructive':ti,ab,kw OR 'chronic

obstructive lung disease':ti,ab,kw OR

coad:ti,ab,kw OR copd:ti,ab,kw OR 'chronic

obstructive airway disease':ti,ab,kw OR 'chronic

obstructive pulmonary disease':ti,ab,kw OR

'airflow obstruction, chronic':ti,ab,kw OR

'chronic airflow obstruction':ti,ab,kw OR

'chronic respiratory diseases':ti,ab,kw

**# Web of Science Search Strategy (v0.1)**

# Database: Web of Science Core Collection

# Entitlements:

- WOS.IC: 1993 to 2023

- WOS.CCR: 1985 to 2023

- WOS.SCI: 1975 to 2023

- WOS.AHCI: 1975 to 2023

- WOS.BHCI: 2005 to 2023

- WOS.BSCI: 2005 to 2023

- WOS.ESCI: 2018 to 2023

- WOS.ISTP: 1990 to 2023

- WOS.SSCI: 1965 to 2023

- WOS.ISSHP: 1990 to 2023

# Searches:

1: Pulmonary Disease, Chronic Obstructive (Topic) OR Chronic Obstructive Lung Disease (Topic) OR COAD (Topic) OR COPD (Topic) OR Chronic Obstructive Airway Disease (Topic) OR Chronic Obstructive Pulmonary Disease (Topic) OR Airflow Obstruction, Chronic (Topic) OR Chronic Airflow Obstruction (Topic) OR chronic obstructive lung disease (Topic) OR chronic airflow obstruction (Topic) OR chronic airway obstruction (Topic) OR chronic obstructive bronchopulmonary disease (Topic) OR chronic obstructive lung disorder (Topic) OR chronic obstructive pulmonary disorder (Topic) OR chronic obstructive respiratory disease (Topic) OR chronic pulmonary obstructive disease (Topic) OR chronic pulmonary obstructive disorder (Topic) OR lung chronic obstructive disease (Topic) OR lung disease, chronic obstructive (Topic) OR obstructive chronic lung disease (Topic) OR obstructive chronic pulmonary disease (Topic) OR obstructive lung disease, chronic (Topic) OR pulmonary disease, chronic obstructive (Topic) OR pulmonary disorder, chronic obstructive (Topic) OR chronic respiratory diseases (Topic) OR Bronchiectasis (Topic) OR Bronchiectases (Topic) OR Bronchiectasis (Topic) OR bronchiectasia (Topic) OR bronchoectasia (Topic) OR Lung Diseases, Interstitial (Topic) OR Diffuse Parenchymal Lung Disease (Topic) OR Interstitial Lung Disease (Topic) OR Lung Disease, Interstitial (Topic) OR Pneumonia, Interstitial (Topic) OR Interstitial Pneumonia (Topic) OR Pneumonias, Interstitial (Topic) OR Pneumonitis, Interstitial (Topic) OR Interstitial Pneumonitides (Topic) OR Interstitial Pneumonitis (Topic) OR Pneumonitides, Interstitial (Topic) OR interstitial lung disease (Topic) OR diffuse interstitial pneumopathy (Topic) OR diffuse parenchyma lung disease (Topic) OR diffuse parenchymal pulmonary disease (Topic) OR diffuse parenchymal pulmonary disorder (Topic) OR interstitial lung disorder (Topic) OR interstitial pneumopathy (Topic) OR interstitial pulmonary disease (Topic) OR interstitial pulmonary disorder (Topic) OR pneumopathy, interstitial (Topic) Date Run: Tue Nov 14 2023 19:06:37 GMT+0800 (中国标准时间) Results: 184714

2: Telerehabilitation (Topic) OR Tele rehabilitation (Topic) OR Remote Rehabilitation (Topic) OR Rehabilitation, Remote (Topic) OR Virtual Rehabilitation (Topic) OR Rehabilitation, Virtual (Topic) OR Telerehabilitation (Topic) OR e-rehabilitation (Topic) OR Telenursing (Topic) OR Telenursing (Topic) OR virtual nursing (Topic) OR Telemedicine (Topic) OR Tele Referral (Topic) OR Virtual Medicine (Topic) OR Medicine, Virtual (Topic) OR Tele Intensive Care (Topic) OR Tele ICU (Topic) OR Mobile Health (Topic) OR Health, Mobile (Topic) OR mHealth (Topic) OR Telehealth (Topic) OR EHealth (Topic) OR telemedicine (Topic) OR Virtual Reality (Topic) OR Reality, Virtual (Topic) OR Educational Virtual Realities (Topic) OR Reality, Educational Virtual (Topic) OR Virtual Realities, Educational (Topic) OR Instructional Virtual Realities (Topic) OR Realities, Instructional Virtual (Topic) OR Reality, Instructional Virtual (Topic) OR Virtual Realities, Instructional (Topic) OR virtual reality (Topic) OR Videogame (Topic) OR computergame (Topic) OR television game (Topic) OR TV games (Topic) OR Home-based (Topic) OR computerized treatment (Topic) OR computerized training (Topic) OR computer-assisted (Topic) Date Run: Tue Nov 14 2023 19:12:20 GMT+0800 (中国标准时间) Results: 282817

3: #2 AND #1 Date Run: Tue Nov 14 2023 19:14:15 GMT+0800 (中国标准时间) Results: 2553
