# Supplementary material for: The effects of telerehabilitation on physiological function and disease symptom for patients with chronic respiratory disease: a systematic review and meta-analysis
Source: BMC Pulm Med. 2024 Jun 28;24:305. doi: 10.1186/s12890-024-03104-8 (PMC11212271; doi:10.1186/s12890-024-03104-8)
Supplement: Supplementary file 2 — Supplementary Material 2: Funnel plot and Sensitivity Analysis. [file 12890_2024_3104_MOESM2_ESM.docx]

**Supplementary Materials 2:** Results of Funnel plots and Sensitivity Analysis

**Funnel plot Results of 6MWD**


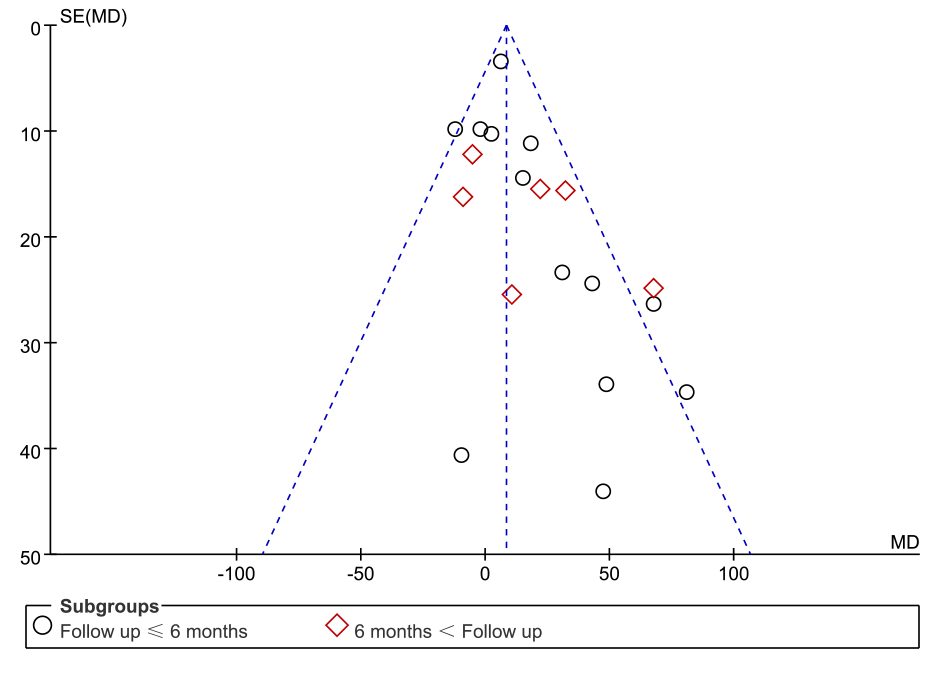


**Funnel plot Results of mMRC**

**
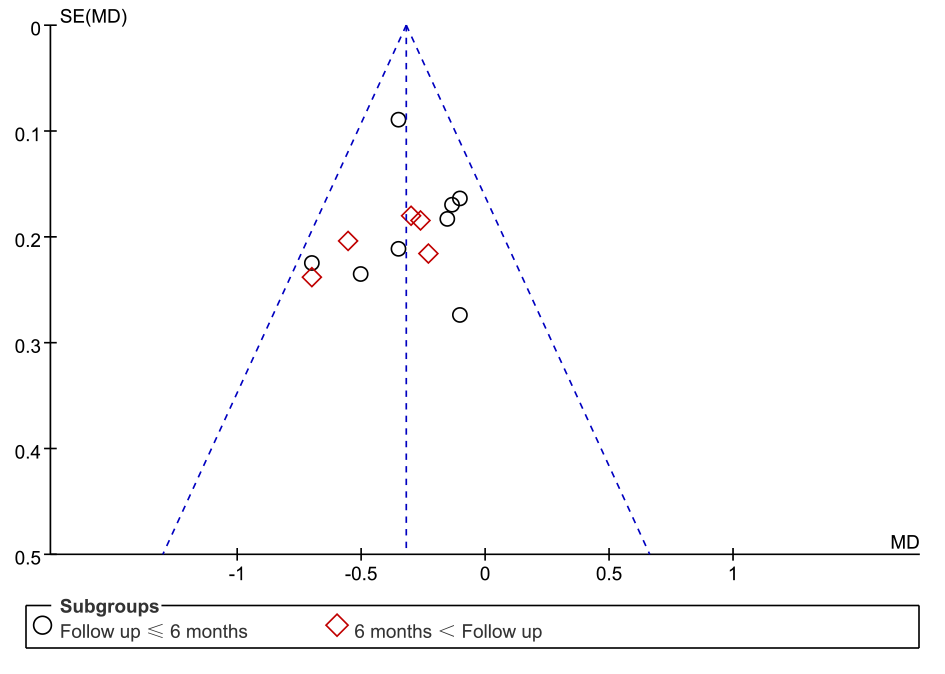
**

**Funnel plot Results of SGRQ**

**ACTIVITY**

**
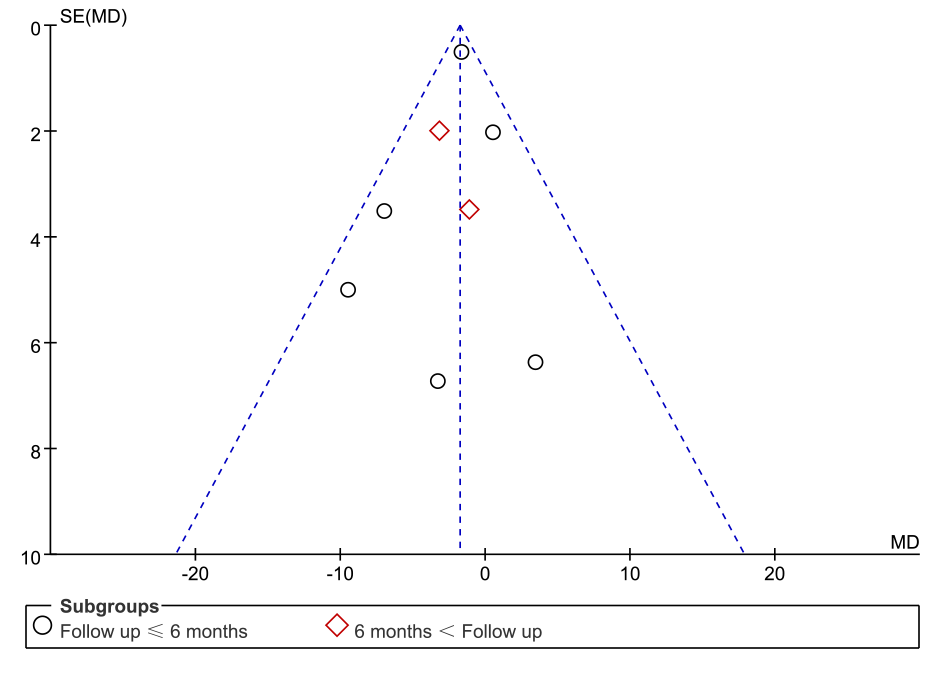
**

**IMPACT**

**
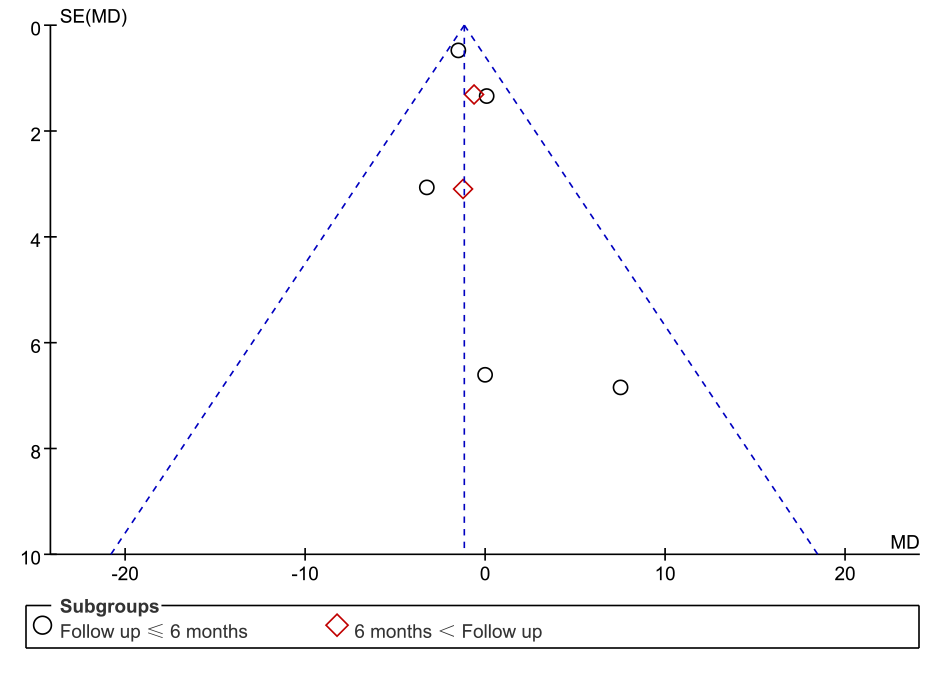
**

**SYMPTOM**

**
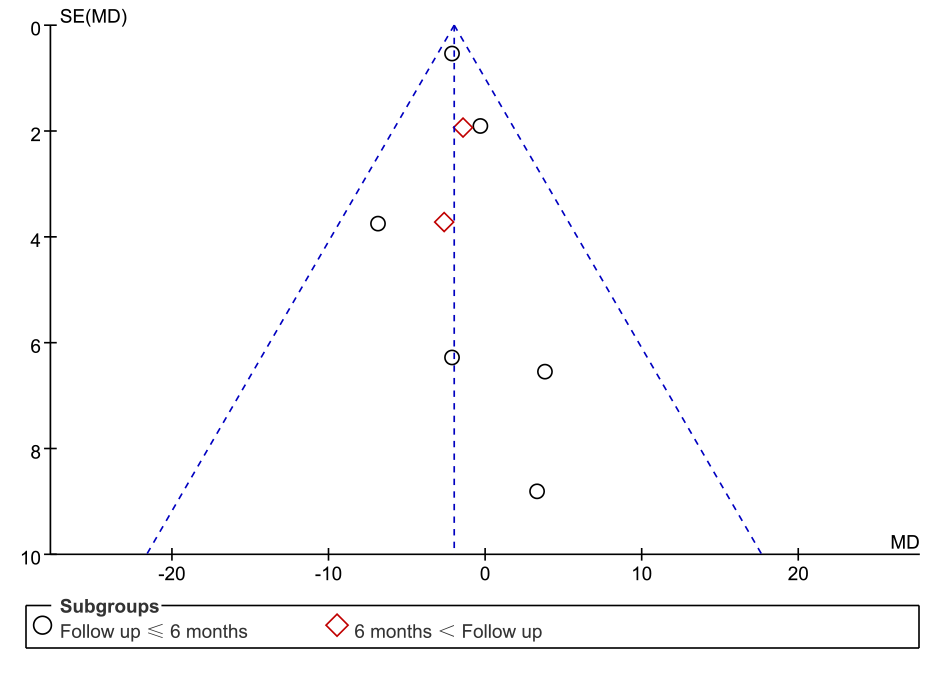
**

**Funnel plot Results of CAT**

**
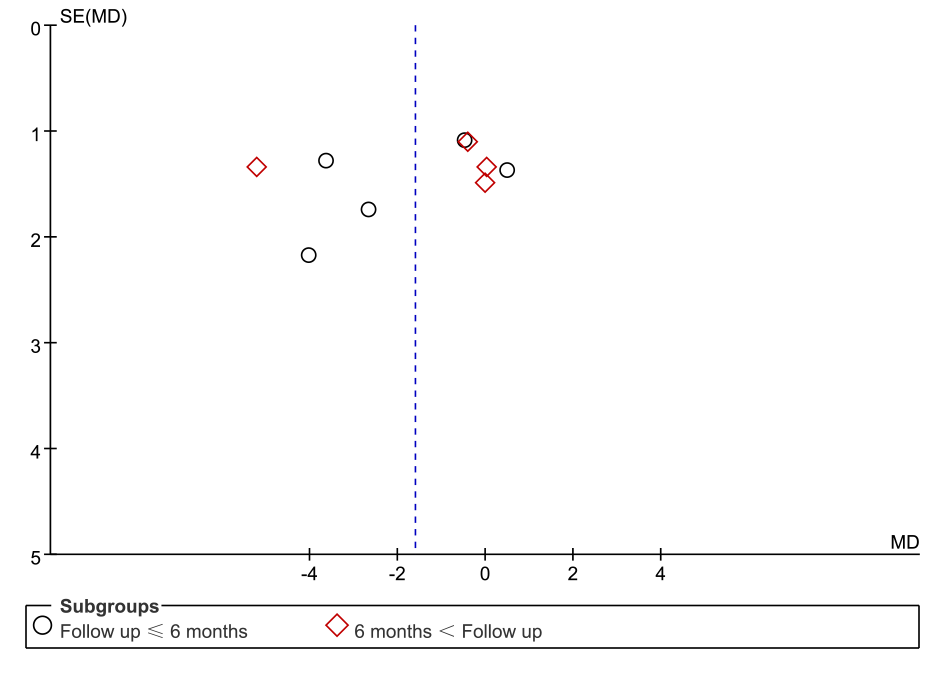
**

**Funnel plot Results of Pulmonary Function**

**
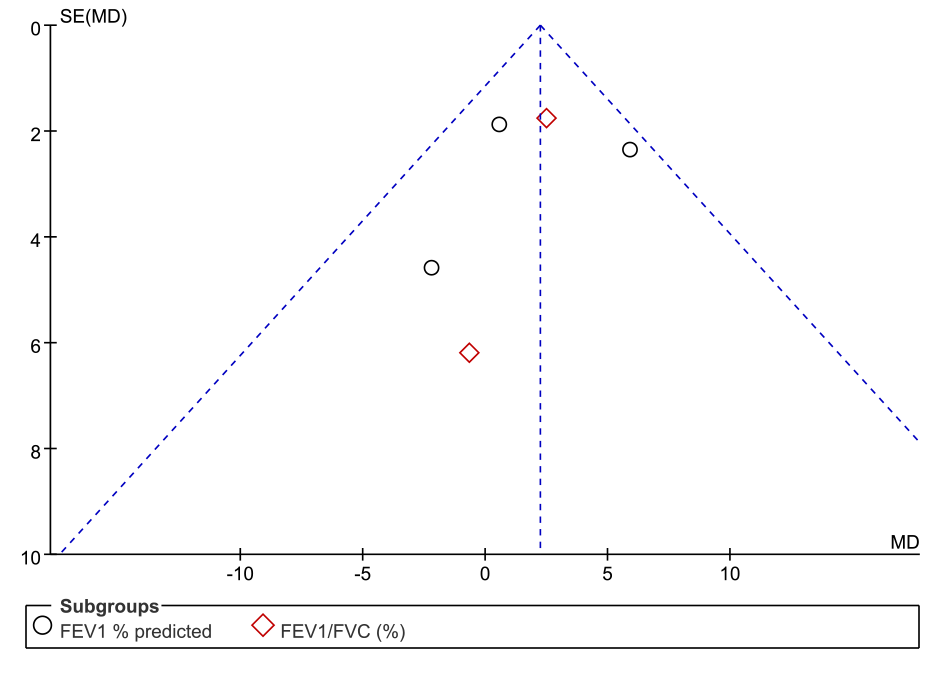
**

**Sensitivity Analysis Results of 6MWD**

**Follow up≤6 months**

**
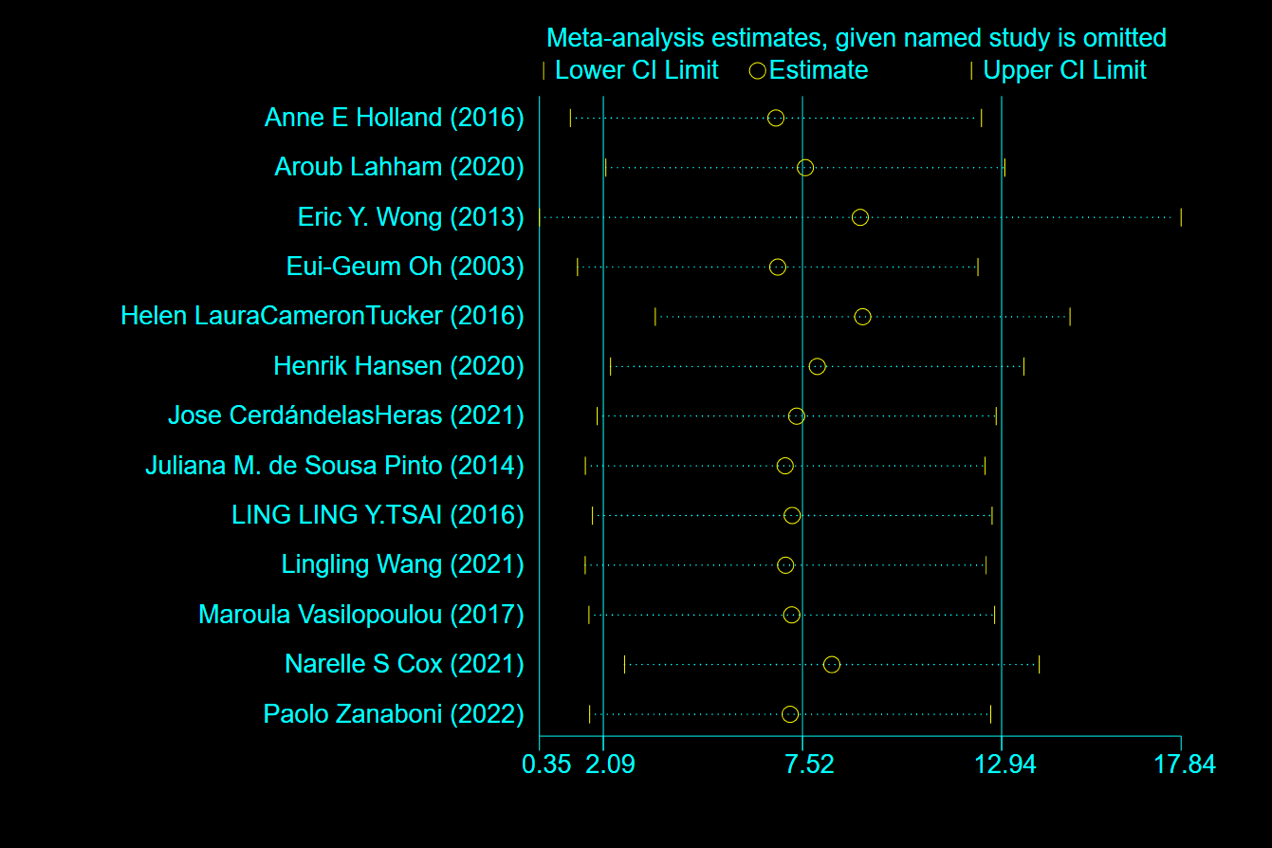
**

**Follow up > 6 months**

**
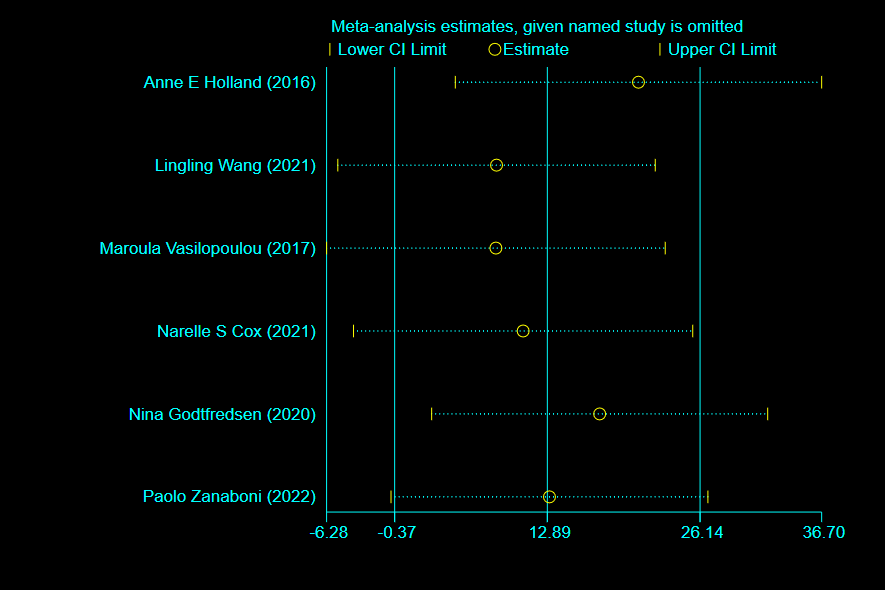
**

**Sensitivity Analysis Results of mMRC**

**Follow up≤6 months**

**
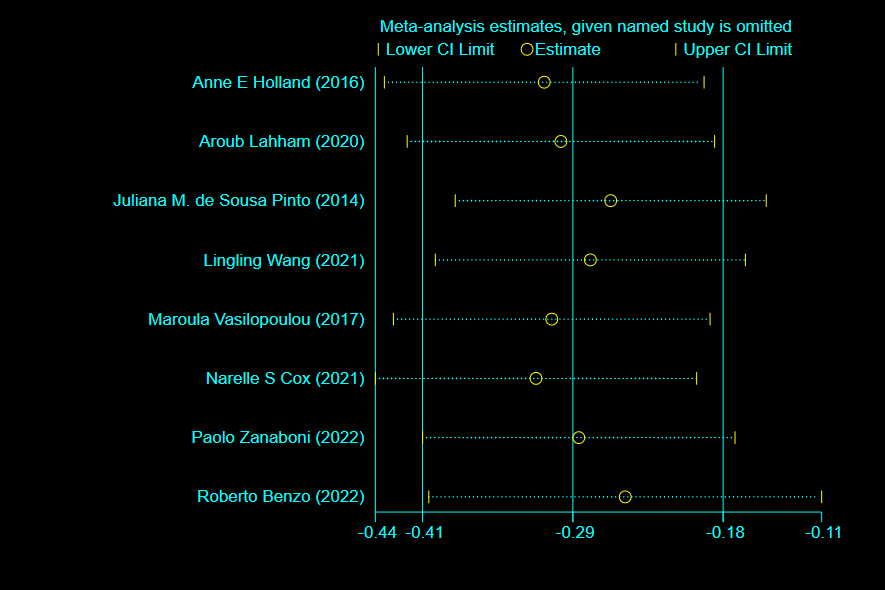
**

**Follow up>6 months**

**
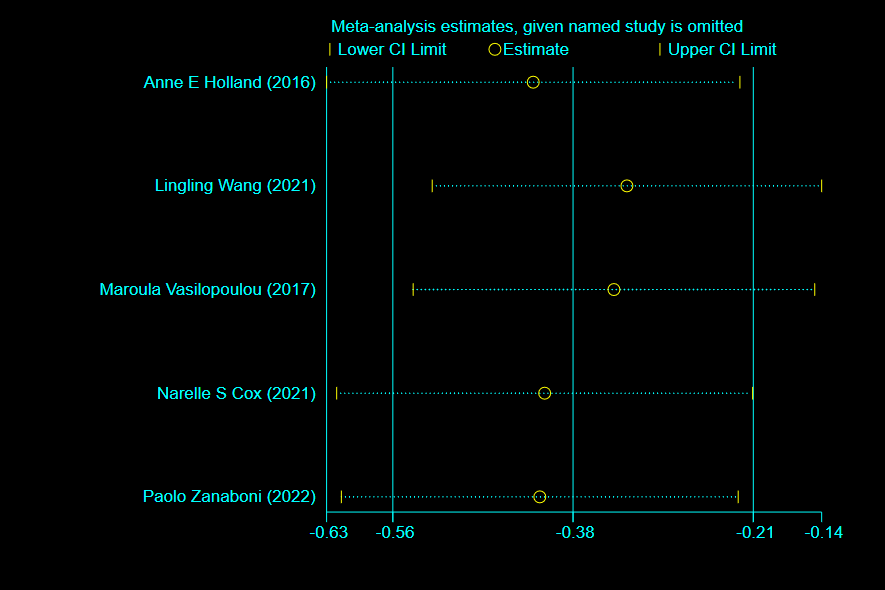
**

**Sensitivity Analysis Results of SGRQ**

**ACTIVITY**

**
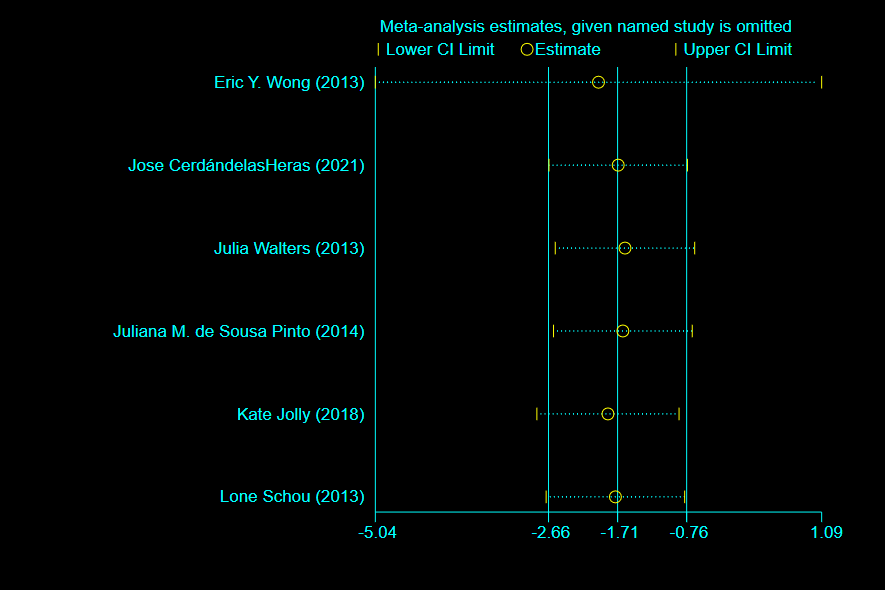
**

**IMPACT**

**
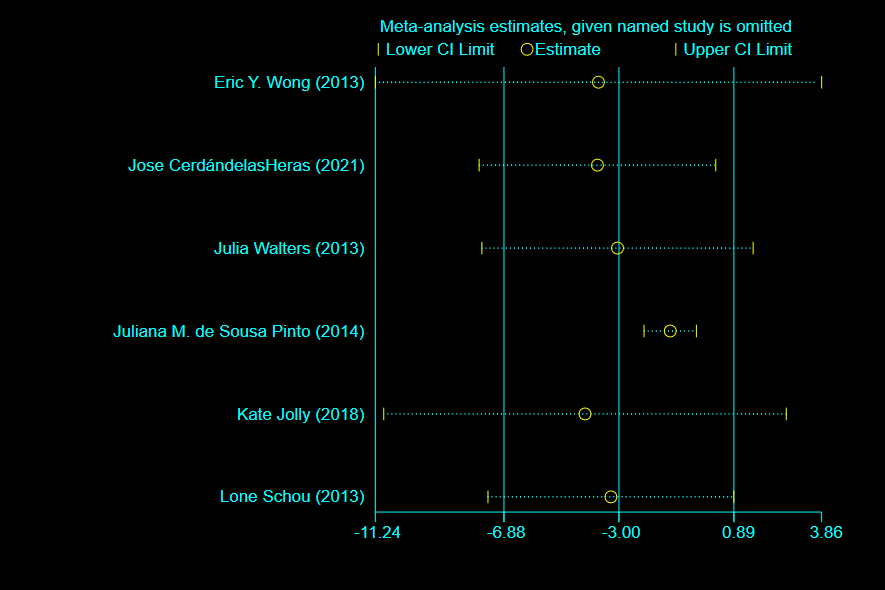
**

**SYMPTOM**

**
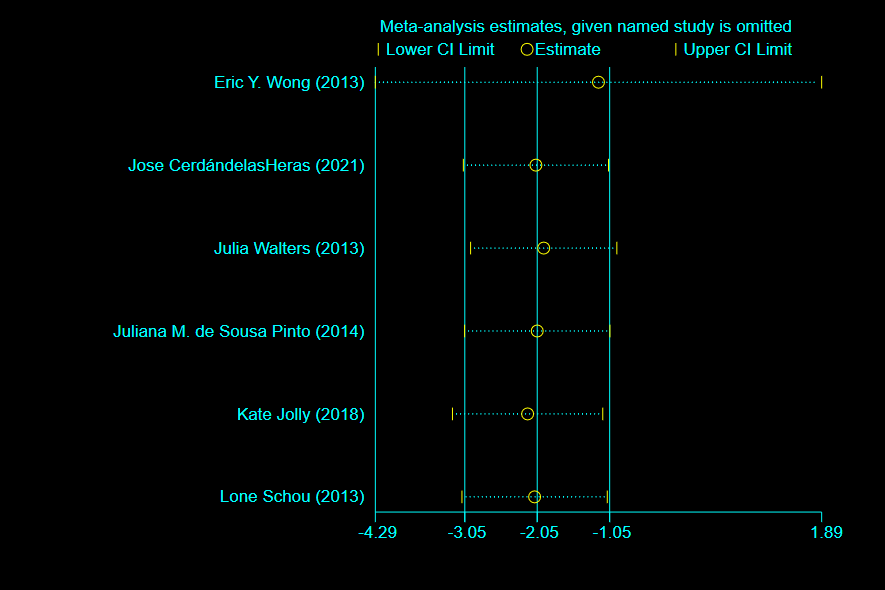
**

**Sensitivity Analysis Results of CAT**

**
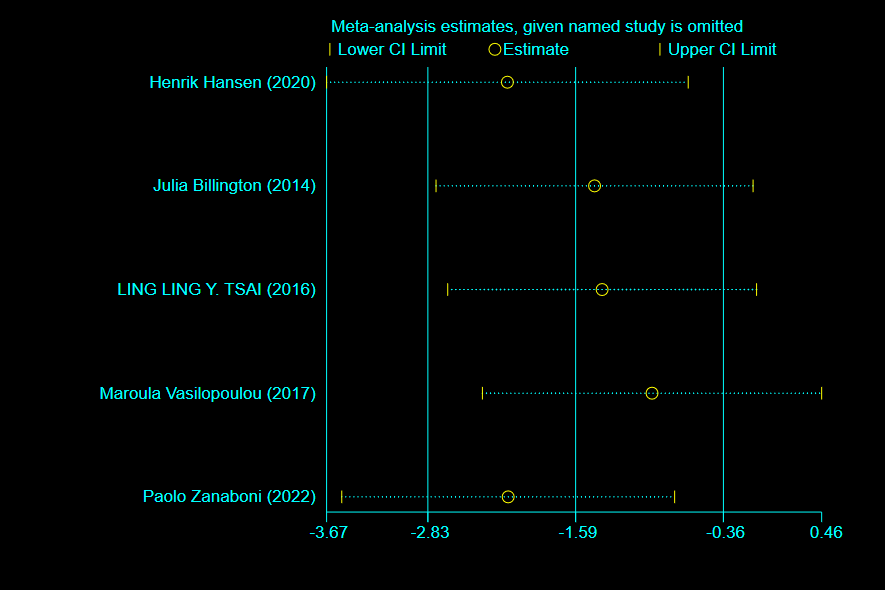
**

**Sensitivity Analysis Results of HADS**

**
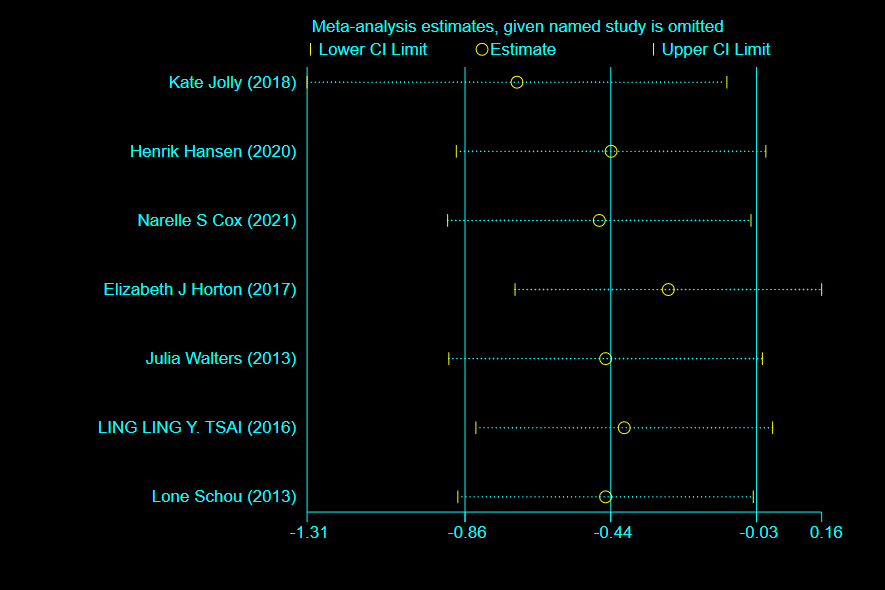
**
